# Protecting double Holliday junctions ensures crossing over during meiosis

**DOI:** 10.1038/s41586-025-09555-1

**Published:** 2025-09-24

**Authors:** Shangming Tang, Sara Hariri, Regina Bohn, John E. McCarthy, Jennifer Koo, Mohammad Pourhosseinzadeh, Emerald Nguyen, Natalie Liu, Christopher Ma, Hanyu Lu, Monica Lee, Neil Hunter

**Affiliations:** 1 Howard Hughes Medical Institute, University of California Davis, Davis, CA USA; 2 Department of Microbiology and Molecular Genetics, University of California Davis, Davis, CA USA; 3 Department of Biochemistry and Molecular Genetics, University of Virginia, Charlottesville, VA USA; 4 Department of Molecular and Cellular Biology, University of California, Davis, Davis, CA USA

**Keywords:** Development, Meiosis, Cohesion, Genomic instability

## Abstract

Chromosomal linkages formed through crossover recombination are essential for the accurate segregation of homologous chromosomes during meiosis^1^. The DNA events of recombination are linked to structural components of meiotic chromosomes^2^. Imperatively, the biased resolution of double Holliday junction (dHJ) intermediates into crossovers^3,4^ occurs within the synaptonemal complex (SC), the meiosis-specific structure that mediates end-to-end synapsis of homologues during the pachytene stage^5,6^. However, the role of the SC in crossover-specific dHJ resolution remains unclear. Here we show that key SC components function through dependent and interdependent relationships to protect dHJs from aberrant dissolution into non-crossover products. Conditional ablation experiments reveal that cohesin, the core of SC lateral elements, is required to maintain both synapsis and dHJ-associated crossover recombination complexes (CRCs) during pachytene. The SC central region transverse-filament protein is also required to maintain CRCs. Reciprocally, the stability of the SC central region requires the continuous presence of CRCs effectively coupling synapsis to dHJ formation and desynapsis to resolution. However, dHJ protection and CRC maintenance can occur without end-to-end homologue synapsis mediated by the central element of the SC central region. We conclude that local ensembles of SC components are sufficient to enable crossover-specific dHJ resolution to ensure the linkage and segregation of homologous chromosomes.

## Abstract (web summary)

Conditional ablation experiments show that key components of the synaptonemal complex protect double Holliday junction recombination intermediates to ensure their resolution into crossover products, which are required for accurate chromosome segregation during meiosis.

## Main

**Extended Data Fig. 1:**
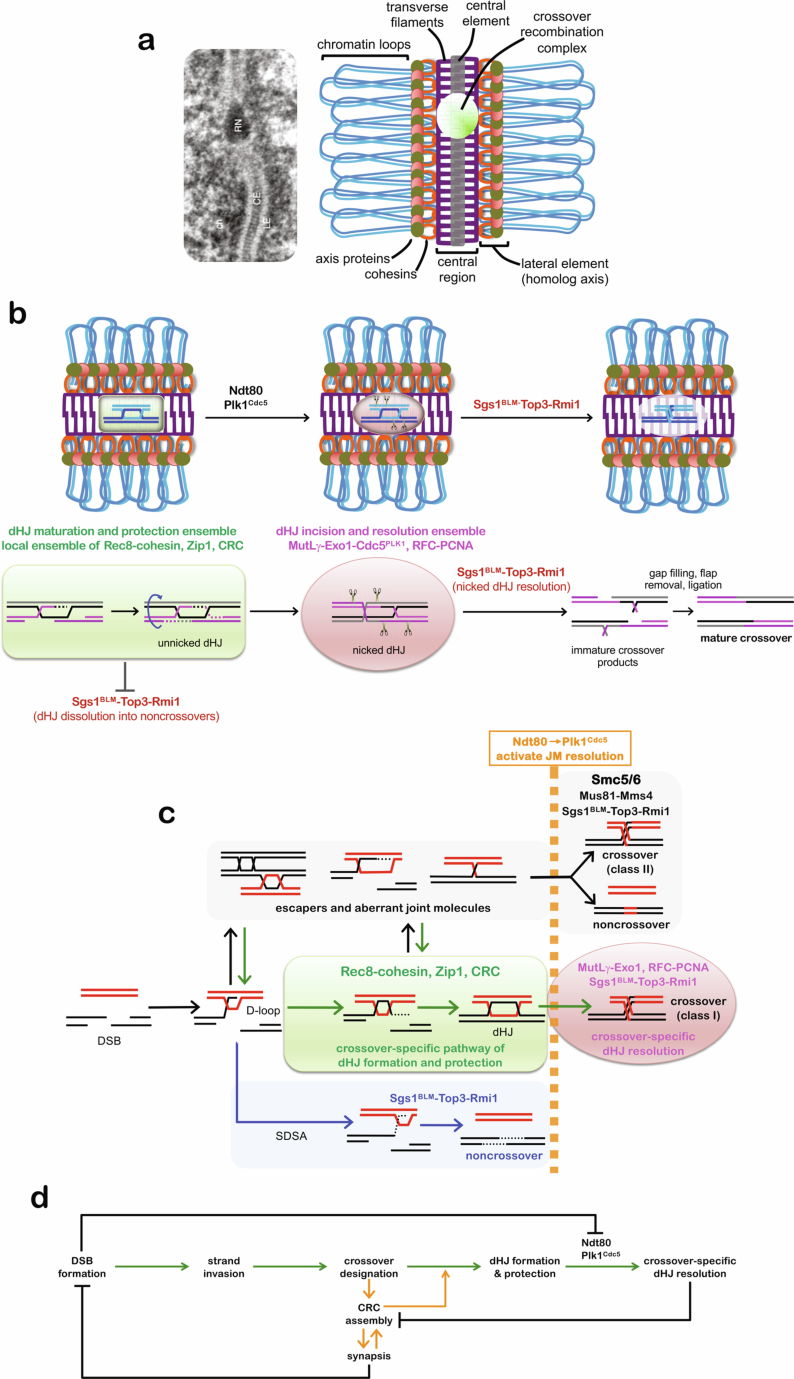
Summary and Model. **a**, Electron micrograph of SC from *Blaps cribrosa* and schematic highlighting the key features of pachytene-stage chromosomes. Ch, chromatin; LE, lateral element; CE, central element, RN, recombination nodule (the site of a crossover recombination complex, CRC). **b**, Rec8-cohesin, Zip1, and CRCs constitute local dHJ maturation and protection ensembles that facilitates dHJ formation and protects unnicked dHJs from being dissolved into non-crossovers by the STR/BLM complex. dHJ protection must be maintained until Ndt80-dependent expression of polo-like kinase Plk1^Cdc5^ triggers crossover-specific resolution via a two-step mechanism: (i) strand-specific nicking of dHJs by the MutLγ endonuclease and associated factors^4^; (ii) resolution of nicked dHJs by the BLM/STR complex. Placement of the nicks, on both sides of the two Holliday junctions but distal to the exchange points, specifies a crossover outcome. **c**, Pathways of joint-molecule resolution during meiotic recombination. Resected DSB ends undergo homology search and DNA strand exchange to form nascent displacement loops (D-loops) as common precursors to all pathways. Non-crossover fated D-loops are unwound by BLM/STR to effect synthesis-dependent strand annealing (SDSA), independently of Plk1^Cdc5^. D-loops that are designated a crossover fate mature into dHJs. In this class-I pathway, Plk1^Cdc5^ triggers two-step crossover-specific resolution as described in **b**. All other joint-molecule structures are resolved via a Smc5/6-dependent pathway that is also dependent on Plk1^Cdc5^ and yields class-II crossovers and non-crossovers. **d**, Dependencies that help ensure crossing over between homologs. At designated crossover sites, CRC assembly promotes and maintains dHJs and synapsis. In turn, synapsis globally attenuates DSB formation^55^, thereby diminishing the DNA-damage kinase signaling that inhibits Ndt80 activity^65^. Ndt80-dependent expression of Plk1^Cdc5^ then activates crossover-specific dHJ resolution, CRCs disassemble, and desynapsis ensues. The image in **a** was adapted from ref. ^66^ (Springer Nature Limited).

During meiotic prophase I, cohesin complexes connect sister chromatids and mediate their organization into linear arrays of chromatin loops tethered to a common axis^2,5,7–9^. These cohesin-based axes define interfaces for the pairing and synapsis of homologous chromosomes that culminates in the formation of SCs. An SC is a tripartite structure comprising the two juxtaposed homologue axes, now called lateral elements, connected by a central lattice of transverse filaments^5,6^. Extension of this lattice to achieve full synapsis requires an additional central element complex^5,6,10^ (Extended Data Fig. 1a). Meiotic recombination facilitates pairing and synapsis between homologous chromosomes and then connects them through crossing over. These connections are necessary for accurate segregation during the first meiotic division^1^. To this end, the DNA events of recombination are physically and functionally linked to underlying chromosome structures^2^. The protein complexes that catalyse DNA double-strand breaks (DSBs) and subsequent strand exchange are tethered to homologue axes. The ensuing joint molecule intermediates and their associated recombination complexes interact with the central region of the SC. A subset of recombination events is assigned a crossover fate with a tightly regulated distribution to ensure that each chromosome pair receives at least one^2^. At designated sites, nascent joint molecules mature into dHJs that then undergo biased resolution specifically into crossovers^3,4^. These steps occur in the context of the SC central region and associated CRCs. The post-synapsis roles of SC components in crossing over remain unclear, particularly whether the SC functions after dHJ formation to facilitate crossover-specific resolution.

## Cohesin and crossover-biased dHJ resolution

**Fig. 1:**
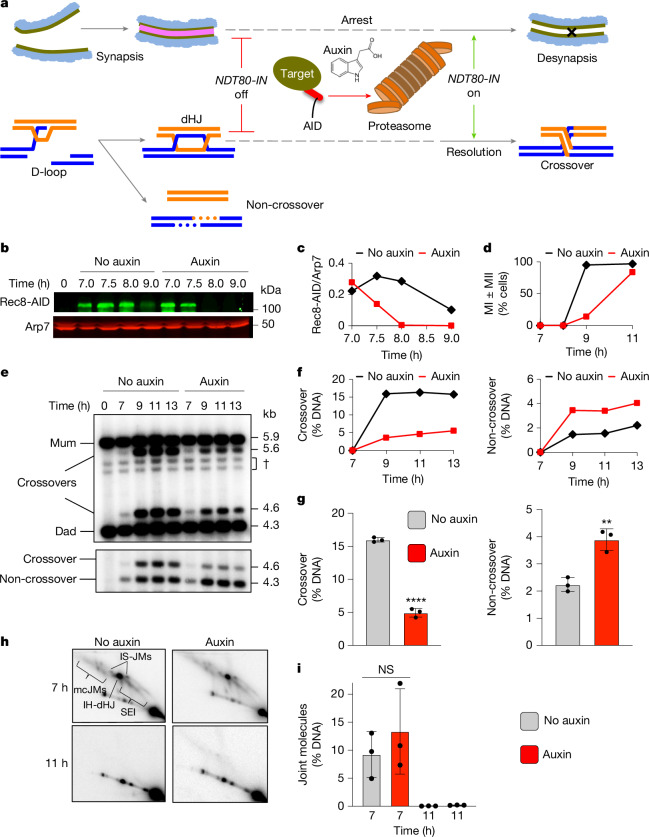
Rec8–cohesin is required for crossover-specific dHJ resolution. **a**, Schematic of the experimental strategy. Top, homologue synapsis with chromatin (blue), homologue axes (green) and the SC central region (pink). The X on the far right of the cartoon indicates a crossover formed after Ndt80 is expressed. Middle, cell synchronization at dHJ resolution transition using the inducible *NDT80-IN* allele and conditional degradation of target proteins using the AID system. Bottom, DNA events of meiotic recombination. Only the two chromosomes engaged in recombination are shown. **b**, Western blot analysis of Rec8-AID from subcultures (released from arrest) with or without the addition of auxin at 7 h. Arp7 was used as the loading control. **c**, Quantification of Rec8-AID levels from the experiment in **b**. **d**, Quantification of nuclear divisions (MI ± MII, cells with two and four nuclei) from *REC8-AID* subcultures with or without auxin. **e**, Representative 1D gel Southern blot analysis of crossover (top) and non-crossover (bottom) formation at the *HIS4::LEU2* recombination hotspot from *REC8-AID* subcultures with or without auxin. The dagger symbol indicates cross-hybridizing background bands. **f**, Quantification of crossover and non-crossover levels from the experiments in **e**. **g**, Quantification of the final levels of crossovers and non-crossovers at 11 h from *REC8-AID* subcultures with or without auxin. Mean ± s.d. of three independent experiments; ****P < 0.0001, **P = 0.0038, two-tailed unpaired *t*-test. **h**, Representative 2D gel Southern blot analysis of joint molecules from *REC8-AID* subcultures with or without auxin and before and after release from arrest at 7 and 11 h, respectively. The top-left panel highlights joint molecule species: IH-dHJ, inter-homologue double Holliday junction; IS-JMs, inter-sister joint molecules; mc-JMs, 3-chromatid and 4-chromatid joint molecules; SEI, single-end invasion. **i**, Quantification of total joint molecule levels from experiments in **h**. Mean ± s.d. of three independent experiments; NS, not significant, *P* = 0.4561, two-tailed unpaired *t*-test.

**Extended Data Fig. 2:**
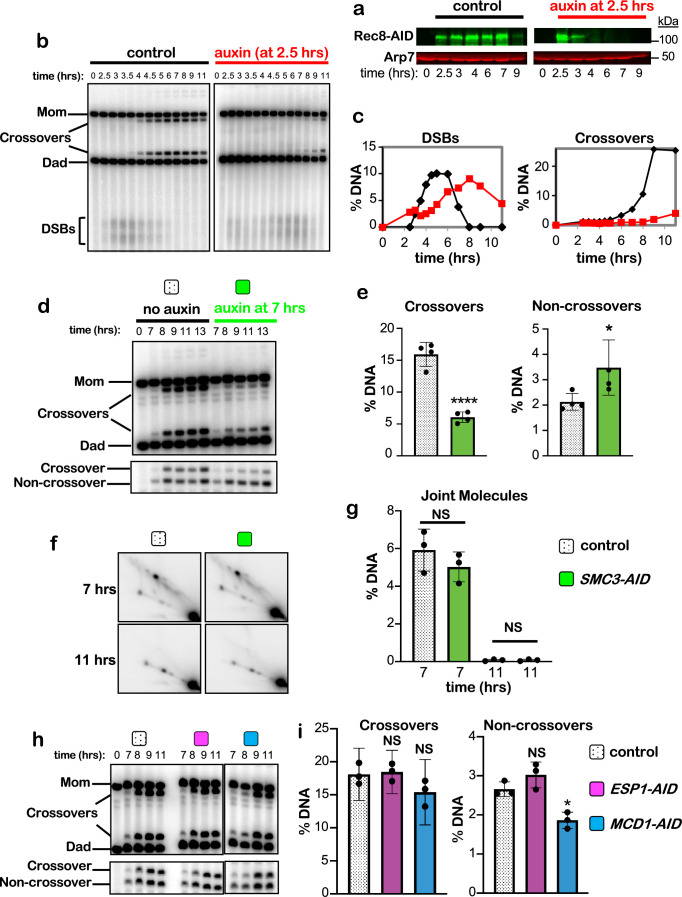
Early functions of Rec8 and roles of Smc3, Kleisin Mdc1^Rad21^, and Separase Esp1 in crossover-specific dHJ resolution. **a**, Western analysis of early Rec8-AID degradation following addition of auxin at 2.5 h. **b**, 1D-gel Southern analysis of DSBs and crossovers following early degradation of Rec8-AID. **c**, Quantification of the DSB and crossover level from the Southern blot shown in C. **d**, Representative 1D-gel Southern analysis of crossover (upper panel) and non-crossover (lower panel) formation from *SMC3-AID* subcultures with or without auxin. **e**, Quantification of final levels of crossovers and non-crossovers at 13 h from *SMC3-AID* subcultures with or without auxin (mean ± SD, n = 4 independent experiments, ****P < 0.0001, NS, not significant *P* = 0.0561, two-tailed unpaired *t*-test). **f**, Representative 2D gel Southern analysis of joint molecules from *SMC3-AID* subcultures with or without auxin, before and after release from arrest at 7 and 11 h, respectively. **g**, Quantification of total joint molecule levels from experiments represented in panel **f** (mean ± SD, n = 3 independent experiments). NS, not significant, *P* = 0.3167 for levels at 7hrs; NS, not significant, *P* > 0.9999 for levels at 11hrs; two-tailed unpaired *t*-test. **h**, Representative 1D-gel Southern analysis of crossover (upper panel) and non-crossover (lower panel) formation in control, *ESP1-AID* (with auxin) or *MCD1-AID* (with auxin) strains. **i**, Final levels of crossovers and non-crossovers at 11 h from the indicated strains (mean ± SD, n = 3 independent experiments). *P = 0.0146; NS, not significant, *P* = 0.9610 (control vs. *ESP1-AID*, crossovers), *P* = 0.1793 (control vs. *MCD1-AID* crossovers), *P* = 0.2073 (control vs. *ESP1-AID*, non-crossovers), Dunnett’s multiple comparisons test, control.

To address the role of SC lateral elements in crossover-specific dHJ resolution, the cohesin core was ablated using the auxin-inducible degron (AID) system^11^ to conditionally degrade Rec8 (the meiosis-specific kleisin subunit) at the time of dHJ resolution (Fig. 1a). Real-time inactivation of Rec8–cohesin circumvents severe early meiotic defects of cohesin mutants in the formation and processing of DSBs^12,13^ (Extended Data Fig. 2a–d). In all experiments, cell cultures were synchronized at the resolution transition using an oestradiol-inducible allele of *NDT80* (*NDT80-IN*) that reversibly arrests cells at the pachytene stage, in which chromosomes are fully synapsed and dHJs are poised for resolution^14^.

Auxin and oestradiol were simultaneously added to degrade Rec8-AID while releasing cells from pachytene arrest and triggering dHJ resolution (Fig. 1b–d). Without auxin, Rec8-AID levels remained high until cells had completed meiotic divisions (meiosis I (MI) + meiosis II (MII)). By contrast, with auxin, Rec8-AID was completely degraded within 60 min and meiotic divisions were delayed by around 2 h (Fig. 1c,d). Crossing over at the *HIS4::LEU2* DSB hotspot^15^ was reduced by 70% after Rec8-AID degradation and was accompanied by a 77% increase in non-crossover products (Fig. 1e–g; note that our assay reports a subset of non-crossover gene-conversion products and not absolute levels of non-crossovers^15^). The opposing changes in levels of crossovers and non-crossovers and the comparable kinetics of their formation with and without auxin (Fig. 1e,f) suggest that dHJs are efficiently resolved when Rec8-AID is degraded, but their resolution fate is reversed. This inference was confirmed by 2D gel electrophoresis and Southern blotting (Fig. 1h,i). Thus, Rec8-based cohesin is required after dHJ formation to facilitate crossover-specific resolution. Degradation of a core cohesin subunit, Smc3, confirmed that the cohesin complex, not just Rec8, is required for this process (Extended Data Fig. 2e–h). However, the mitotic kleisin Mcd1 (known as RAD21 in other species) was not required for crossover-specific dHJ resolution, a result that indicates that this function is specific to Rec8–cohesin (Extended Data Fig. 2i,j). The separase Esp1 also did not influence crossover-specific resolution (Extended Data Fig. 2i), a finding consistent with previous observations^16^ showing that a separase-resistant allele of Rec8 does not affect crossing over.

## Distinct functions of cohesin and the Smc5/6 complex

**Fig. 2:**
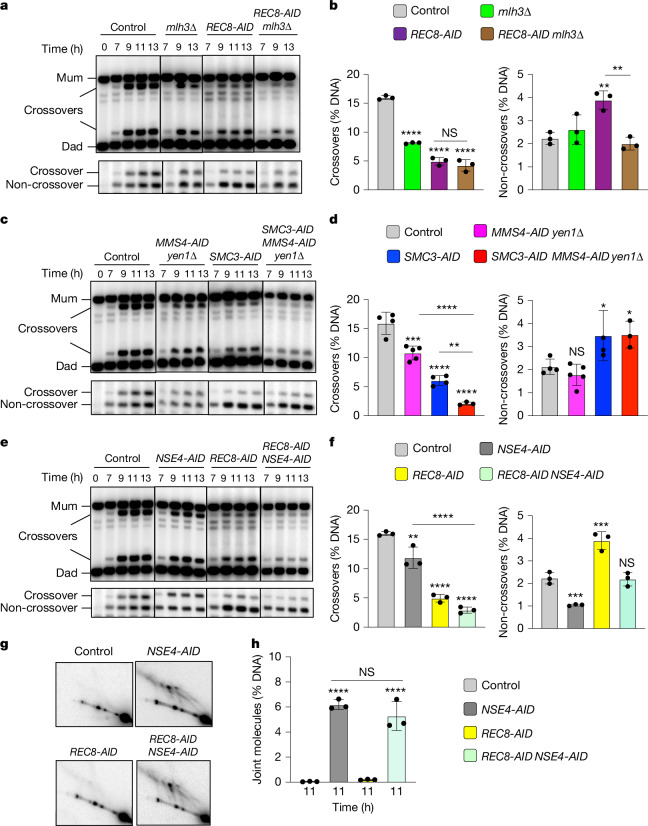
Rec8–cohesin and Smc5/6 define distinct pathways of joint molecule resolution. In all experiments shown in this figure, cells were released from *NDT80-IN* arrest. **a**, Representative 1D gel Southern blot analysis of crossover (top) and non-crossover (bottom) formation in control (*REC8-AID* no auxin), *mlh3*Δ, *REC8-AID* (with auxin) and *REC8-AID* *mlh3*Δ (with auxin) strains. **b**, Final levels of crossovers and non-crossovers at 13 h from the indicated strains. Mean ± s.d. of three independent experiments. Statistical comparisons are with the control unless indicated otherwise. Dunnett’s multiple comparisons test, ****P < 0.0001, NS, *P* = 0.5281, **P = 0.0034 (*REC8-AID* versus control), **P = 0.0026 (*REC8-AID* versus *REC8-AID* *mlh3*Δ). **c**, Representative 1D gel Southern blot analysis of crossover (top) and non-crossover (bottom) formation in control, *MMS4-AID* *yen1*Δ (with auxin), *SMC3-AID* (with auxin) and *SMC3-AID* *MMS4-AID* *yen1*Δ (with auxin) strains. **d**, Final levels of crossovers and non-crossovers at 11 h from the indicated strains. Mean ± s.d. of *n* = 4 control, *n* = 5 *MMS4-AID* *yen1*Δ, *n* = 4 *SMC3-AID* and *n* = 3 *SMC3-AID MMS4-AID* *yen1*Δ independent experiments. Statistical comparisons are with the control unless indicated otherwise. For crossovers, Tukey’s multiple comparisons test was performed; for non-crossovers, Dunnett’s multiple comparisons test was performed. ****P < 0.0001, ***P = 0.0003, **P = 0.0066, *P = 0.0385 (control versus *SMC3-AID*), *P = 0.0488 (control versus *SMC3-AID* *MMS4-AID* *yen1*Δ), NS, *P* = 0.7806. **e**, Representative 1D gel Southern blot analysis of crossover (top) and non-crossover (bottom) formation in control, *NSE4-AID* (with auxin), *REC8-AID* (with auxin) and *REC8-AID* *NSE4-AID* (with auxin) strains. **f**, Final levels of crossovers and non-crossovers at 11 h from experiments in **c**. Mean ± s.d. of three independent experiments. Statistical comparisons are with the control unless indicated otherwise. Dunnett’s multiple comparisons test, ****P < 0.0001, **P = 0.0049, ***P = 0.0022 (non-crossovers *NSE4-AID* versus control), ***P = 0.0002 (non-crossovers *REC8-AID* versus control); NS, *P* = 0.9955 (non-crossovers *REC8-AID* *NSE4-AID* versus control). **g**, Representative Southern blot 2D gel images at 11 h from no-auxin control, *NSE4-AID* (with auxin), *REC8-AID* (with auxin) and *REC8-AID* *NSE4-AID* (with auxin). **h**, Quantification of total joint molecule levels from the experiments in **e**. Mean ± s.d. of three independent experiments. Tukey’s multiple comparison test, ****P < 0.0001, NS, *P* = 0.3320.

Two classes of meiotic crossovers are distinguished by their dependencies on enzymes that resolve joint molecules. Class I crossovers depend on the crossover-specific dHJ resolvase defined by the endonuclease MutLγ (a complex of Mlh1 and Mlh3)^3,4,17–19^. By contrast, minority class II crossovers require structure-selective endonucleases, primarily Mus81–Mms4 (known as MUS81–EME1 in other species)^3,20–22^. Epistasis analysis revealed that cohesin and MutLγ act in the same crossover resolution pathway (Fig. 2a,b), whereas Mus81–Mms4 acts in a parallel pathway (Fig. 2c,d; in these experiments, Mms4-AID was degraded at the resolution transition in a *yen1*Δ background, in which the backup resolvase Yen1 is deleted). Crossover levels were indistinguishable after Rec8-AID degradation in the presence or absence of MutLγ (in cells with a *mlh3*Δ mutation; Fig. 2b). However, in contrast to the *REC8-AID* single mutant in which non-crossovers were increased (Fig. 1g), non-crossovers in *REC8-AID* *mlh3*Δ strains were reduced to control levels (Fig. 2b). This result suggests that MutLγ influences gene conversion of the central polymorphism used to detect non-crossovers^15^. That is, non-crossover products that contain unrepaired heteroduplex DNA or undergo restoration of the central polymorphism, which are undetectable with our assay, are increased in *REC8-AID* *mlh3*Δ cells.

**Extended Data Fig. 3:**
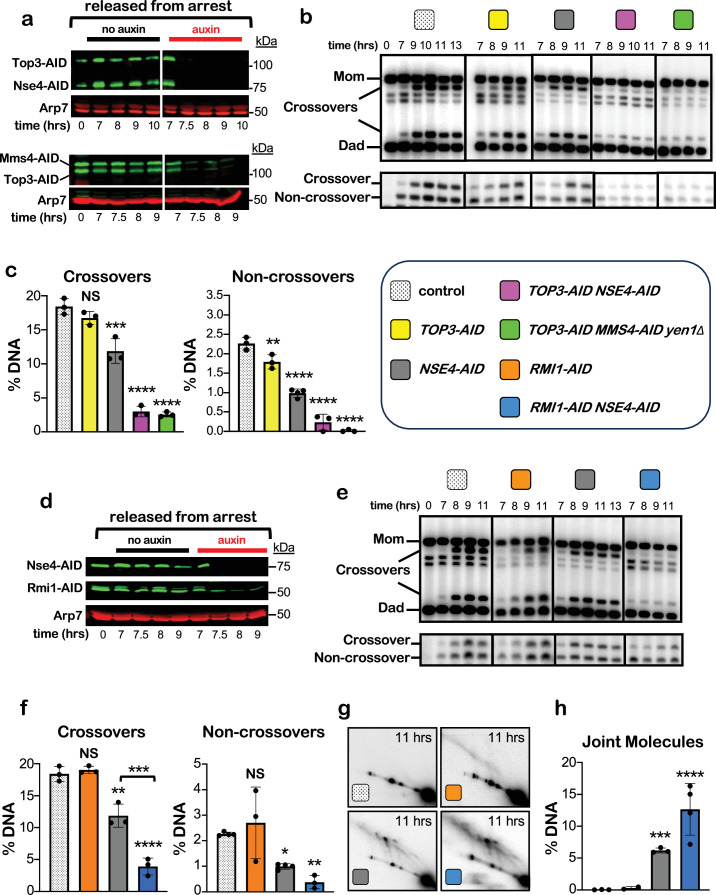
Sgs1-Top3-Rmi1 and Smc5/6 mediate essentially all joint-molecule resolution. **a**, Western blot analysis of Top3-AID and Nse4-AID co-degradation in cells released from pachytene-arrest and addition of auxin at 7 h. **b**, Representative 1D-gel Southern analysis of crossover (upper panel) and non-crossover (lower panel) formation in control, *TOP3-AID* (with auxin), *NSE4-AID* (with auxin), *TOP3-AID NSE4-AID* (with auxin) and *TOP3-AID NSE4-AID yen1Δ* (with auxin) following release from pachytene arrest. **c**, Final levels of crossovers and non-crossovers at 11 h from experiments represented in **b**. (mean ± SD, n = 3 independent experiments). Statistical comparisons with control: ****P < 0.0001, ***P = 0.0002 (*NSE4-AID* vs. control, crossovers), **P = 0.0251 (*TOP3-AID* vs. control, non-crossovers), NS not significant *P* = 0.4157 (*TOP3-AID* vs. control, crossovers), Tukey’s multiple comparisons test. **d**, Western blot analysis of RMI1-AID and Nse4-AID co-degradation in cells released from pachytene-arrest and addition of auxin at 7 h. **e**, Representative 1D-gel Southern analysis of crossover (upper panel) and non-crossover (lower panel) formation in control, *RMI1-AID* (with auxin), *NSE4-AID* (with auxin) and *RMI1-AID NSE4-AID* (with auxin) following release from pachytene arrest. **f**, Final levels of crossovers and non-crossovers at 11 h from experiments represented in **b**. (mean ± SD, n = 3 independent experiments for control, *RMI1-AID*, and *RMI1-AID NSE4-AID*; n = 4 for *NSE4-AID*). Statistical comparisons with control: ****P < 0.0001, ***P = 0.0003 (*NSE4*-*AID* vs. *RMI1-AID NSE4-AID*, crossovers), **P = 0.0012 (*NSE4*-*AID* vs. control, crossovers),**P = 0.0086 (*RMI1-AID NSE4-AID* vs. control, non-crossovers), *P = 0.0474 (*NSE4-AID* vs. control, non-crossovers), NS, not significant *P* = 0.9395 (*RMI1*-*AID* vs. control, crossovers), *P* = 0.7225 (*RMI1-AID* vs. control, non-crossovers,), Tukey’s multiple comparisons test. **g**, Representative 2D gel Southern analysis of joint molecules from control, *RMI1-AID, NSE4-AID and RMI1-AID NSE4-AID* subcultures with auxin after release from arrest at 11 h. **h**, Quantification of total joint molecule levels from experiments represented in panel **g** (mean ± SD, n = 3 independent experiments for control, *RMI1-AID*, and *NSE4-AID*; n = 4 for *RMI1-AID NSE4-AID*). NS, not significant *P* = 0.8596, ***P = 0.0002, ****P < 0.0001, Dunnett’s multiple comparisons test).

Mus81–Mms4 works in conjunction with a second SMC complex, Smc5/6 (refs. ^21–24^). Consistently, degradation of Nse4-AID (an essential subunit of Smc5/6) during the resolution transition reduced crossing over to the same extent as when Mus81–Mms4 was inactivated by degrading Mms4-AID (Fig. 2c–f). Although non-crossovers were not reduced when Mus81–Mms4 was inactivated, Nse4-AID degradation also reduced non-crossovers by 2.1-fold (Fig. 2e,f). This finding indicates that Smc5/6 controls an additional, Mus81–Mms4-independent pathway of non-crossover formation, most probably through the regulation of a subpopulation of the Sgs1–Top3–Rmi1 complex (Extended Data Figs. 1 and 3).

**Extended Data Fig. 4:**
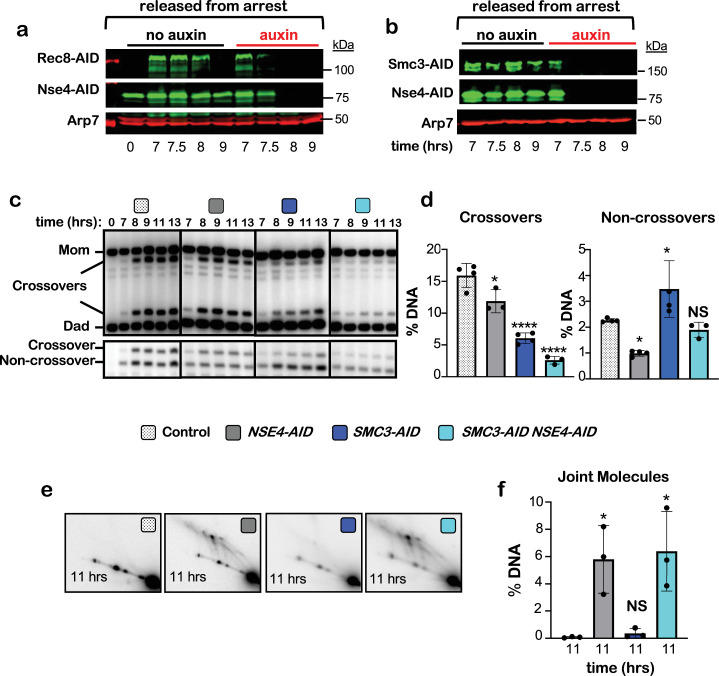
Cohesin and the Smc5/6 complex act in parallel pathways of joint-molecule resolution. **a**, Western blot images of Smc3-AID Nse4-AID co-degradation following auxin addition at 7 h (corresponds to experiments in Fig. 2e–g). Arp7 is a loading control. **b**, Western blot images of Rec8-AID Nse4-AID co-degradation following auxin addition at 7 h. **c**, Representative 1D-gel Southern analysis of crossover (upper panel) and non-crossover (lower panel) formation in control, *NSE4-AID* (with auxin), *SMC3-AID* (with auxin), and *SMC3-AID NSE4-AID* (with auxin) strains. **d**, Final levels of crossovers and non-crossovers at 11 h from the indicated strains (mean ± SD, n = 3 independent experiments for *NSE4-AID*, *SMC3-AID*, and *SMC3-AID NSE4-AID*; n = 4 for control). Statistical comparisons with control unless indicated. For crossovers, Tukey’s multiple comparisons test was performed; for non-crossovers, Dunnett’s multiple comparisons test was performed. ****P < 0.0001; *P = 0.0178 (control vs. *NSE4-AID* crossovers); *P = 0.0277(control vs. *NSE4-AID* non-crossovers), *P = 0.0360 (control vs. *SMC3-AID* non-crossover). NS, not significant, *P* = 0.7563 (control vs. *SMC3-AID NSE4-AID* non-crossovers). **e**, Representative 2D gel Southern analysis of joint molecules from control, *NSE4-AID* (with auxin), *SMC3-AID* (with auxin) and *SMC3-AID NSE4-AID* (with auxin) strains. **f**, Quantification of total joint-molecule levels from the indicated strains. (mean ± SD, n = 3 independent experiments). Tukey’s multiple comparisons test: *P = 0.0269 (control vs. *NSE4-AID*), *P = 0.0162(control vs. *SMC3-AID NSE4-AID*), *P = 0.0207 (SMC3-AID vs. *SMC3-AID NSE4-AID*), NS, not significant *P* = 0.9976 (control vs. *SMC3-AID*).

We next aimed to confirm that Smc5/6 and cohesin act in independent pathways of resolution. Simultaneous degradation of Nse4-AID and Rec8-AID resulted in an additive reduction in crossing over; and non-crossover levels that are the combination of those observed when Nse4-AID and Rec8-AID were individually degraded (Fig. 2e,f). Note that simultaneous degradation of Nse4-AID and Smc3-AID gave analogous results (Extended Data Fig. 4). Further distinguishing these two pathways, a subset of joint molecules remained unresolved when Nse4-AID was degraded alone, whereas resolution remained efficient when cohesin was inactivated through the degradation of either Rec8-AID or Smc3-AID (Figs. 1g,h and 2g,h and Extended Data Fig. 4e,f). These results show that Smc5/6 is essential for the resolution of a subset of joint molecules into both crossovers and non-crossovers. By contrast, Rec8–cohesin promotes crossover-specific dHJ resolution but is not required for resolution per se.

## Cohesin, synapsis and crossover complexes

**Fig. 3:**
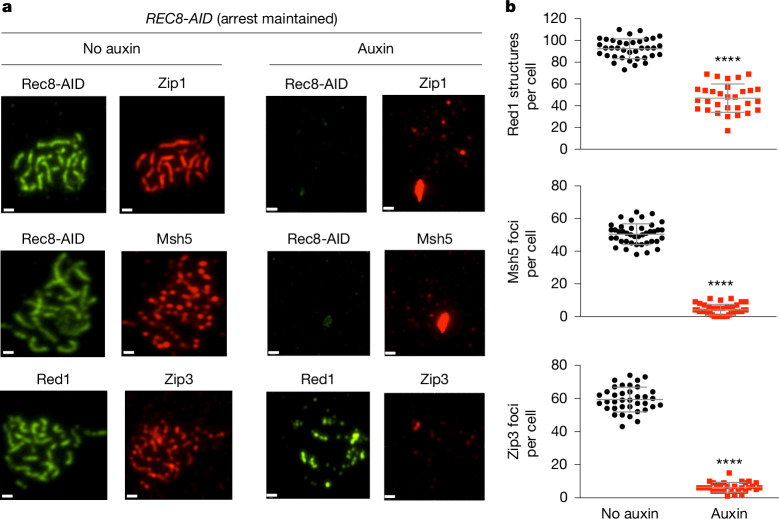
Rec8–cohesin is required to maintain SCs and crossover-specific recombination complexes. **a**, Representative images of surface-spread meiotic nuclei from *REC8-AID* cultures in which pachytene arrest was maintained. Cells were sampled 1 h after the addition of auxin or DMSO vehicle at 7 h and immunostained for the indicated markers. Scale bars, 1 μm. **b**, Quantification of structures immunostained for Red1, Msh5 and Zip3 from experiments in **a**. Mean ± s.d. Red1 staining, *n* = 30 and 37 nuclei; Msh5 staining, *n* = 64 and 57 nuclei; Zip3 staining, *n* = 37 and 31 nuclei (*n* indicates the number of nuclei analysed for the auxin and no-auxin experiments, respectively). Unpaired two-tailed *t*-test. ****P < 0.0001.

To begin to understand how cohesin facilitates crossover-specific dHJ resolution, Rec8-AID was degraded while pachytene arrest was maintained (no induction of *NDT80-IN*). Chromosomes were then analysed by immunostaining for markers of homologue axes (Rec8 and Red1), the SC central region (Zip1) and CRCs (Msh5 and Zip3)^25^ (Fig. 3a). In no-auxin controls, linear Rec8-stained and discontinuous Red1-stained structures colocalized, and synapsed homologues (indicated by lines of Zip1 staining) were decorated with foci of Msh5 and Zip3 (Fig. 3a). One hour after the addition of auxin, Rec8 structures were lost and characteristic features of pachytene chromosomes disappeared. That is, SCs disassembled and Zip1 staining was now confined to a few foci and larger structures resembling polycomplexes that are diagnostic of defective synapsis. Moreover, the numbers of Red1-stained structures were reduced by twofold, a result consistent with the core function of cohesin in organizing homologue axes^26^ and directly recruiting Red1 (ref. ^27^). Furthermore, CRCs were dissociated, as indicated by the loss of Msh5 and Zip3 foci (Fig. 3a,b). Thus, cohesin is required to maintain the integrity of pachytene chromosome structures, a finding consistent with analyses of meiotic cohesin in *Caenorhabditis elegans*^28^.

## Interdependent functions of SC components

**Fig. 4:**
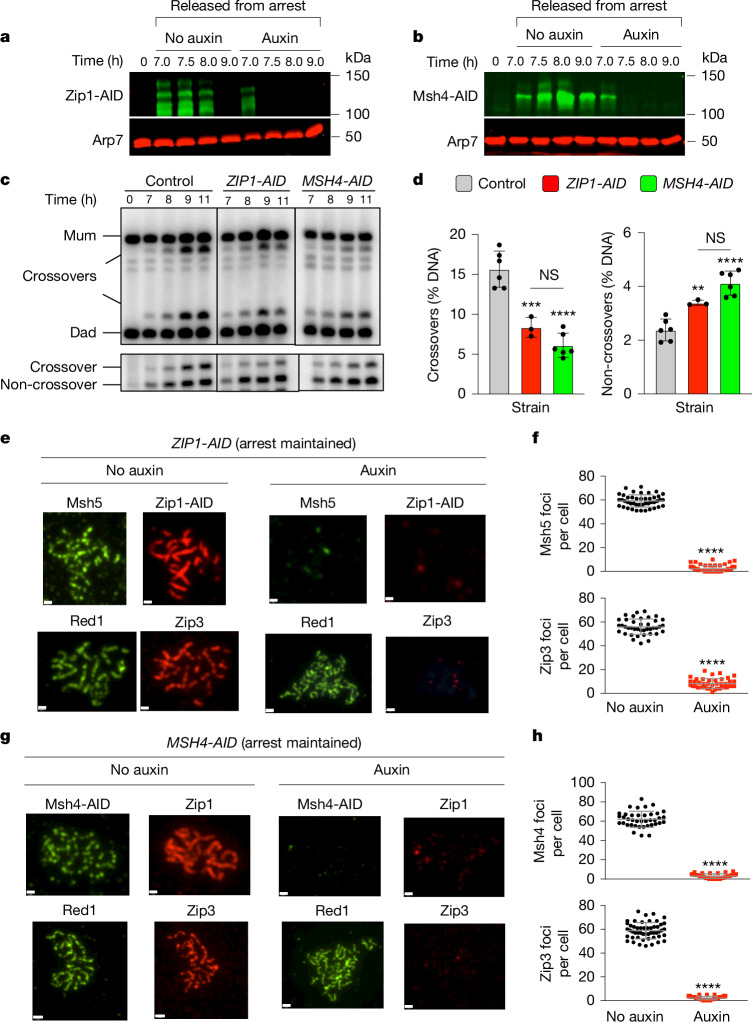
Zip1 and MutSγ are required for crossover-specific dHJ resolution. **a**, Western blot analysis of Zip1-AID from subcultures (released from arrest) with or without the addition of auxin at 7 h. Arp7 was used as the loading control. **b**, Western blot analysis of Msh4-AID from subcultures (released from arrest) with or without the addition of auxin at 7 h. Arp7 was used as the loading control. **c**, Representative 1D gel Southern blot analysis of crossover (top) and non-crossover (bottom) formation in control, *ZIP1-AID* (with auxin) and *MSH4-AID* (with auxin) strains. **d**, Final levels of crossovers and non-crossovers at 11 h from the indicated strains. Mean ± s.d. of *n* = 6 control, *n* = 3 *ZIP1-AID* and *n* = 6 *MSH4-AID* independent experiments. Statistical comparisons are with the control unless indicated otherwise. Tukey’s multiple comparisons test, ****P < 0.0001, ***P = 0.0003, **P = 0.0087, NS, *P* = 0.2321 (*ZIP1-AID* versus *MSH4-AID* for crossover analysis), *P* = 0.0524 (*ZIP1-AID* versus *MSH4-AID* for non-crossover analysis). **e**, Representative images of surface-spread meiotic nuclei from pachytene-arrested *ZIP1-AID* cells sampled 1 h after the addition of auxin or DMSO vehicle at 7 h and immunostained for the indicated markers. **f**, Quantification of foci immunostained for Msh5 and Zip3 from the experiments in **e**. Mean ± s.d. Msh4 staining, *n* = 44 and 62 nuclei; Zip3 staining, *n* = 40 and 41 nuclei (*n* indicates the number of nuclei analysed for the auxin and no-auxin experiments, respectively). Unpaired two-tailed *t-*test, ****P < 0.0001. **g**, Representative images of surface-spread meiotic nuclei from pachytene-arrested *MSH4-AID* cells sampled 1 h after the addition of auxin or DMSO vehicle at 7 h and immunostained for the indicated markers. **h**, Quantification of foci stained for Msh4 and Zip3 from the experiments in **g**. Mean ± s.d. Msh4 staining, *n* = 39 and 35 nuclei; Zip3 staining, *n* = 51 and 49 nuclei (*n* indicates the number of nuclei analysed for the auxin and no-auxin experiments, respectively). Unpaired two-tailed *t*-test, ****P < 0.0001. Scale bars, 1 µm (**e**,**g**).

To discern which cohesin-dependent features of pachytene chromosomes are important for crossover-specific dHJ resolution, the SC transverse-filament protein Zip1 (refs. ^29,30^) and the pro-crossover factor MutSγ (a complex of Msh4 and Msh5) were inactivated as cells were released from pachytene arrest (Fig. 4a,b). MutSγ binds and stabilizes nascent joint molecules to promote homologue synapsis and dHJ formation^31–33^. The degradation of either Zip1-AID or Msh4-AID led to phenotypes similar to those resulting from the loss of cohesin. That is, reduced crossovers and increased non-crossovers (Fig. 4c,d), which indicated that these factors have continued roles after dHJs have formed to maintain their crossover resolution fate.

**Extended Data Fig. 5:**
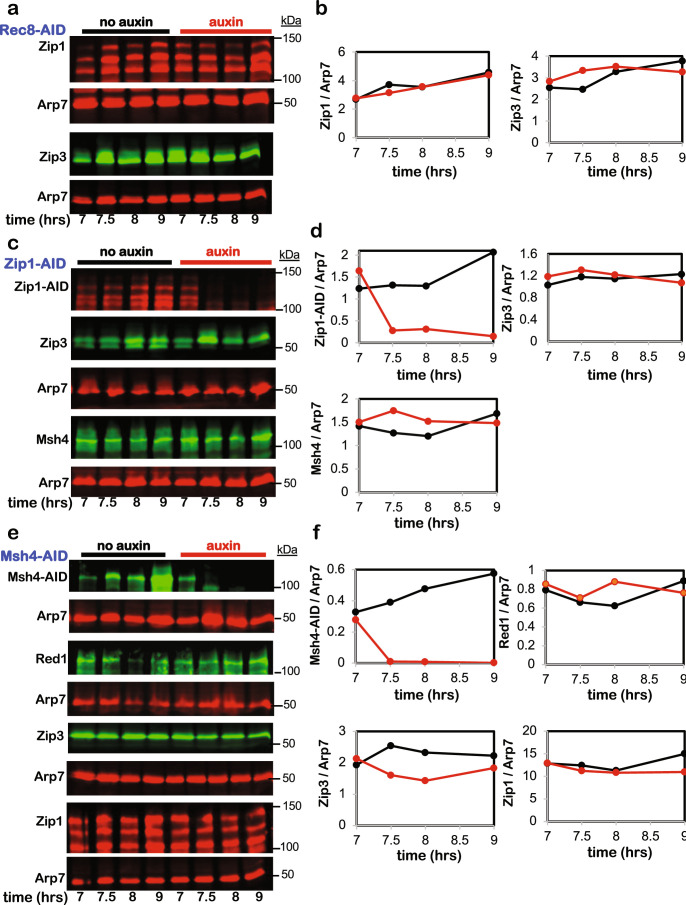
Targeted degradation does not cause off-target effects. **a**, Representative Western blot analysis of Zip1 and Zip3 with or without Rec8-AID degradation following auxin addition at 7 h. **b**, Quantification of Zip1 and Zip3 level from the experiments shown in **a**. **c**, Representative Western blot analysis of Zip3 and Msh4 with or without Zip1-AID degradation following auxin addition at 7 h. **d**, Quantification of Zip1-AID, Zip3, and Msh4 levels from the experiments shown in **c**. **e**, Representative Western blot analysis of Red1, Zip3, and Zip1 with or without Msh4-AID degradation following auxin addition at 7 h. **f**, Quantification of Msh4-AID, Red1, Zip3, and Zip1 level from the experiments shown in **e**. Arp7 is the loading control in all experiments.

These results suggest that cohesin may facilitate crossover-specific dHJ resolution by stabilizing the SC central region, which in turn stabilizes CRCs. This interpretation was tested by immunostaining the chromosomes of pachytene-arrested cells after the degradation of Zip1-AID (Fig. 4e,f) or Msh4-AID (Fig. 4g,h). In budding yeast, full synapsis of the 16-homologue pairs is indicated by around 16 Zip1-stained lines and discontinuous linear Red1-stained structures (Fig. 4e,g, left). When Zip1-AID was degraded, desynapsis ensued, whereby Zip1 lines were lost and the numbers of linear Red1-stained structures approximately doubled, which is indicative of unsynapsed but intact homologue axes (Fig. 4e). As predicted, CRCs marked by Msh5 and Zip3 foci were diminished (Fig. 4e,f). As Zip3 and Msh4 protein levels remained high in these cells (Extended Data Fig. 5), we inferred that CRCs are disassembled when Zip1-AID is degraded. Notably, desynapsis also occurred when Msh4-AID was degraded, which revealed that the maintenance of SCs during pachytene requires the continued presence of CRCs (Fig. 4g). Zip3 foci were also lost when Msh4-AID was degraded. This result highlights the interdependence between Holliday junction binding (MutSγ) and the regulatory (Zip3) components of CRCs (Fig. 4g,h).

## dHJs are protected from aberrant dissolution

**Fig. 5:**
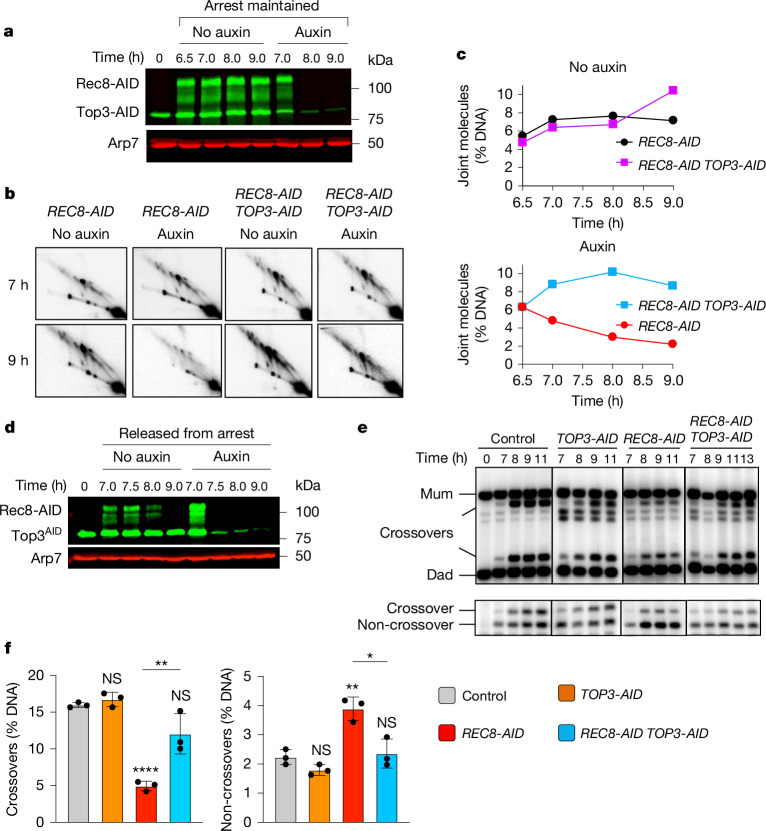
Rec8–cohesin protects dHJs from aberrant resolution mediated by the STR/BLM complex. **a**, Western blot analysis of Top3-AID and Rec8-AID from subcultures with or without the addition of auxin at 7 h and pachytene arrest was maintained. Arp7 was used as the loading control. The degradation-resistant fraction of Top3-AID is mitochondrial. **b**, Representative 2D gel Southern blot analysis of joint molecules from *REC8-AID* and *REC8-AID* *TOP3-AID* strains with and without the addition of auxin. **c**, Quantification of total joint molecule levels from the indicated strains. **d**, Western blot analysis of Top3-AID and Rec8-AID from subcultures with or without the addition of auxin at 7 h with release from pachytene arrest. **e**, Representative 1D gel Southern analysis of crossover (top) and non-crossover (bottom) formation in control, *TOP3-AID* (with auxin), *REC8-AID* (with auxin) and *REC8-AID* *TOP3-AID* (with auxin) strains. **f**, Final levels of crossovers and non-crossovers at 11 h from experiments in **g**. Mean ± s.d. of three independent experiments. Statistical comparisons are with the control unless indicated otherwise. Tukey’s multiple comparisons test, ****P < 0.0001, **P = 0.0018 (*REC8-AID* versus *REC8-AID* *TOP3-AID* for crossover comparison),*P = 0.0489 (control versus *REC8-AID* *TOP3-AID* for crossover comparison), NS, *P* = 0.9219 (control versus *TOP3-AID* for crossover comparison), **P = 0.0021 (control versus *REC8-AID* for non-crossover comparison), **P = 0.0034 (*REC8-AID* versus *REC8-AID* *TOP3-AID* for non-crossover comparison), NS, *P* = 0.4753 (control versus *TOP3-AID* for non-crossover comparison), *P* = 0.9721 (control versus *REC8-AID* *TOP3-AID* for non-crossover comparison).

**Extended Data Fig. 6:**
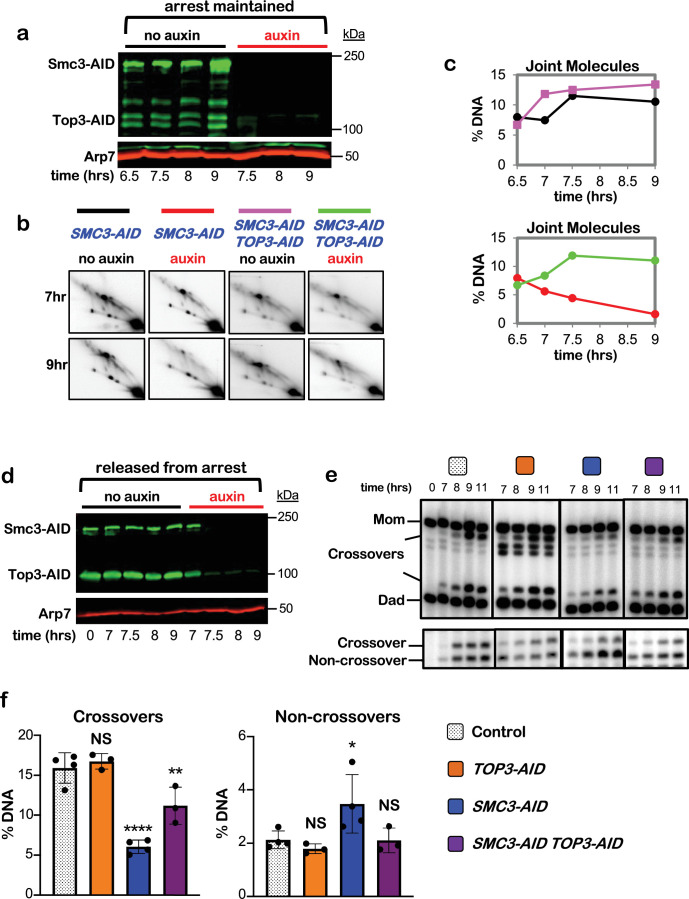
Cohesin protects dHJs from dissolution by the STR/BLM complex. **a**, Western blot analysis of Smc3-AID and Top3-AID co-degradation in pachytene-arrested cells following the addition of auxin at 7 h. Arp7 is a loading control. **b**, Representative 2D gel Southern analysis of joint molecules from subcultures of pachytene arrested *SMC3-AID* and *SMC3-AID TOP3-AID* strains, with or without the addition of auxin. **c**, Quantification of total joint molecules from experiments shown in **b**. **d**, Western blot analysis of Smc3-AID and Top3-AID co-degradation in cells released from pachytene-arrest and addition of auxin at 7 h. Arp7 is used as loading control. **e**, Representative 1D-gel Southern analysis of crossover (upper panel) and non-crossover (lower panel) formation in control, *TOP3-AID* (with auxin), *SMC3-AID* (with auxin) and *SMC3-AID TOP3-AID* (with auxin) following release from pachytene arrest. **f**, Final levels of crossovers and non-crossovers at 11 h from experiments represented in **e** (mean ± SD, n = 3 independent experiments for *TOP3-AID* and *SMC3-AID TOP3-AID*; n = 4 for control and *SMC3-AID*). Statistical comparisons with control: ****P < 0.0001, ** *P* = 0.0038, NS, not significant *P* = 0.9876 (*TOP3-AID* vs. control crossovers), *P = 0.0423. NS, not significant *P* = 0.8549 (*TOP3-AID* vs. control non-crossovers), NS, not significant *P* > 0.9999 (*TOP3-AID SMC3-AID* vs. control non-crossovers), Dunnett’s multiple comparisons test.

dHJs normally remain stable in pachytene-arrested *NDT80-IN* cells because the expression of the polo-like kinase Plk1 (also known as Cdc5), which activates dHJ resolution, requires the transcription factor Ndt80 (refs. ^14,34^). However, dHJ levels decreased by around threefold when Rec8-AID was degraded but pachytene arrest was maintained (Fig. 5b,c). This result implied that pachytene chromosome structures protect dHJs against aberrant resolution by a Plk1-independent resolution activity. A good candidate for this activity is the Sgs1–Top3–Rmi1 (STR) complex, the budding yeast orthologue of the human BLM complex BLM–TOPIIIα–RMI1/2, and a robust decatenase enzyme that can dissolve dHJs specifically into non-crossover products^35–39^. Confirming this prediction, dHJs were stabilized when Rec8-AID and Top3-AID were simultaneously degraded (Fig. 5a–c). Note that the degradation of both Smc3-AID and Top3-AID gave similar results (Extended Data Fig. 6). Moreover, dHJ resolution after Rec8-AID degradation alone produced non-crossover products that did not form when Top3-AID was also degraded (Fig. 5d,e).

**Extended Data Fig. 7:**
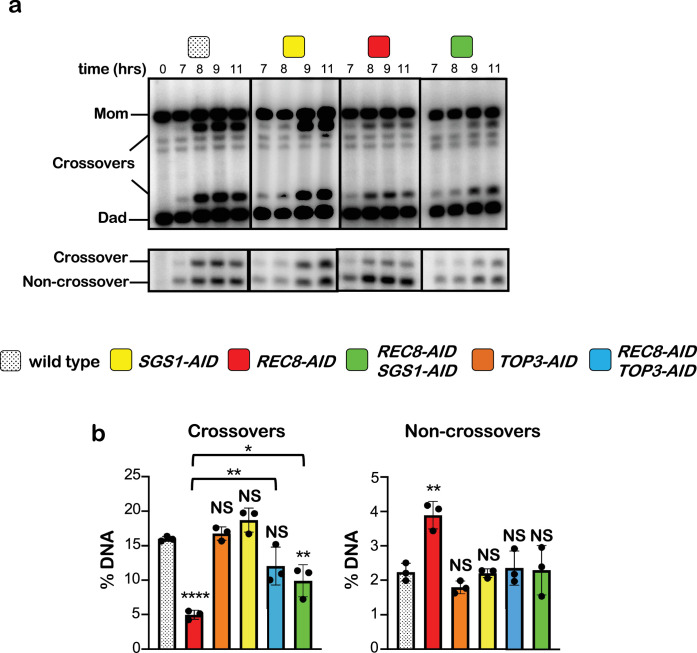
Sgs1-AID degradation partially suppresses the crossover defect resulting from degradation of Rec8-AID. **a**, Representative 1D-gel Southern analysis of crossover (upper panel) and non-crossover (lower panel) formation following release from pachytene arrest in control, *SGS1-AID* (with auxin), *REC8-AID* (with auxin), *REC8-AID SGS1-AID* (with auxin). **b**, Final levels of crossovers and non-crossovers at 11 h from the indicated strains (mean ± SD, n = 3 independent experiments). Data for *TOP3-AID* and *REC8-AID TOP3-AID* from Fig. 5e are shown for comparison. ****P < 0.0001, **P = 0.0037 (*REC8-AID SGS1-AID* vs. control crossovers), **P = 0.0027(*REC8-AID TOP3-AID* vs. *REC8-AID* crossovers), **P = 0.0016 (*REC8-AID* vs. control non-crossovers), *P = 0.0356 (*REC8-AID SGS1-AID* vs. *REC8-AID* crossovers). NS, not significant: *P* = 0.9712 (*TOP3-AID* vs. control crossovers), *P* = 0.2430 (*SGS1-AID* vs. control crossovers), *P* = 0.0563 (*REC8-AID TOP3-AID* vs. control crossovers), *P* = 0.5846 (*TOP3-AID* vs. control non-crossovers), *P* > 0.9999 (*SGS1-AID* vs. control non-crossovers), *P* = 0.9949 (*REC8-AID TOP3-AID* vs. control non-crossovers), *P* = 0.9998 (*REC8-AID SGS1-AID* vs. control non-crossovers), Dunnett’s multiple comparisons tests were used to compare auxin treated cells with control. Tukey’s multiple comparisons tests were used to compare individual treatment cells.

**Extended Data Fig. 8:**
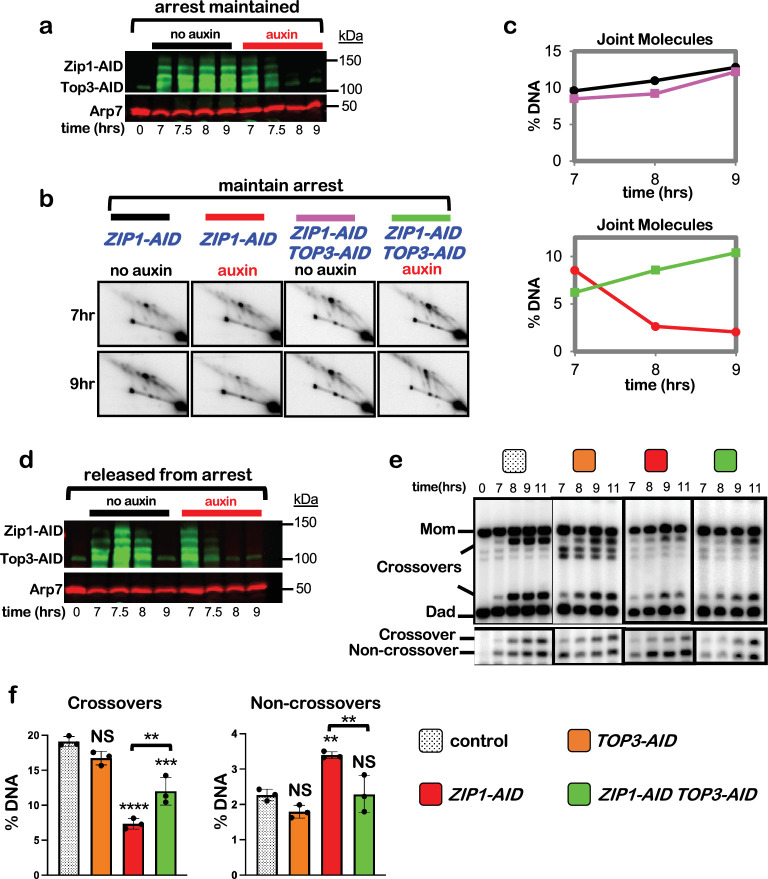
Zip1 protects double Holliday junctions from aberrant resolution mediated by the STR/BLM complex. **a**, Western blot analysis of Zip1-AID and Top3-AID co-degradation in pachytene-arrested cells following the addition of auxin at 7 h. Arp7 is a loading control. **b**, Representative 2D gel Southern analysis of joint molecules from subcultures of pachytene arrested *ZIP1-AID* and *ZIP1-AID TOP3-AID* strains, with or without the addition of auxin. **c**, Quantification of total joint molecules from experiments shown in **b**. **d**, Western blot analysis of Zip1-AID and Top3-AID co-degradation in cells released from pachytene-arrest and addition of auxin at 7 h. **e**, Representative 1D-gel Southern analysis of crossover (upper panel) and non-crossover (lower panel) formation in control, *TOP3-AID* (with auxin), *ZIP1-AID* (with auxin) and *ZIP1-AID TOP3-AID* (with auxin) following release from pachytene arrest. **f**, Final levels of crossovers and non-crossovers at 11 h from experiments represented in **e**. (mean ± SD for 3 independent experiments). Statistical comparisons with control unless stated: ****P < 0.0001, ***P = 0.0005, **P = 0.0071(*ZIP1-AID TOP3-AID* vs. *ZIP1-AID* crossovers), **P = 0.0065(*ZIP1-AID* vs. control non-crossovers), **P = 0.0072 (*ZIP1-AID TOP3-AID* vs. *ZIP1-AID* non-crossovers), NS not significant *P* = 0.1642 (*TOP3-AID* vs. control crossovers), *P* = 0.2717 (*TOP3-AID* vs. control non-crossovers), *P* = 0.9998 (*ZIP1-AID TOP3-AID* vs. control non-crossovers), Tukey’s multiple comparisons tests.

**Extended Data Fig. 9:**
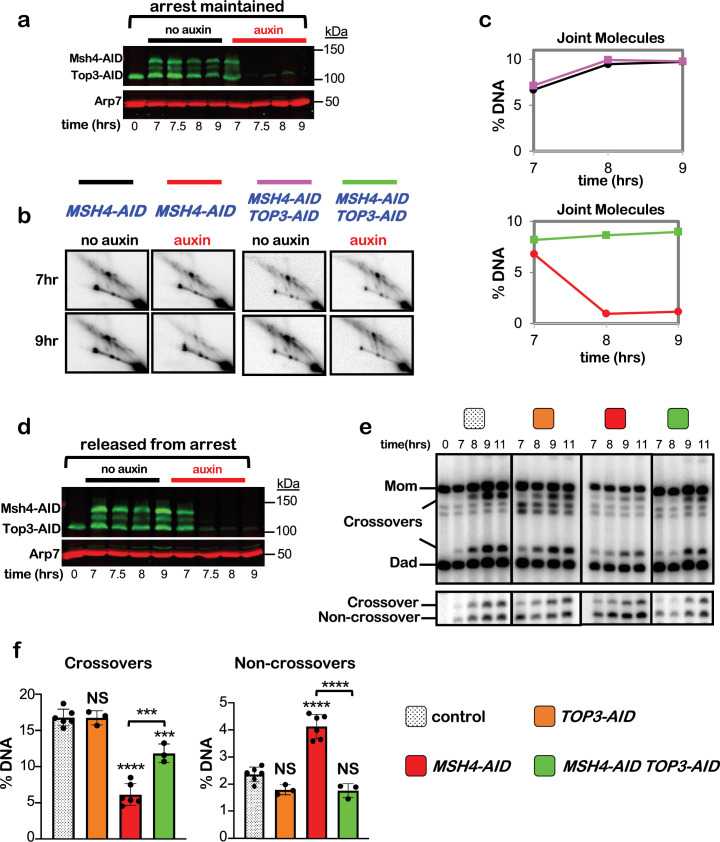
MutSγ protects double Holliday junctions from aberrant resolution mediated by the STR/BLM complex. **a**, Western blot analysis of Msh4-AID and Top3-AID co-degradation in pachytene-arrested cells following the addition of auxin at 7 h. Arp7 is a loading control. **b**, Representative 2D gel Southern analysis of joint molecules from subcultures of pachytene arrested *MSH4-AID* and *MSH4-AID TOP3-AID* strains, with or without the addition of auxin. **c**, Quantification of total joint molecules from experiments shown in **b**. **d**, Western blot analysis of Msh4-AID and Top3-AID co-degradation in cells released from pachytene-arrest and addition of auxin at 7 h. **e**, Representative 1D-gel Southern analysis of crossover (upper panel) and non-crossover (lower panel) formation in control, *TOP3-AID* (with auxin), *MSH4-AID* (with auxin) and *MSH4-AID TOP3-AID* (with auxin) following release from pachytene arrest. **f**, Final levels of crossovers and non-crossovers at 11 hrs from experiments represented in **e**. (mean ± SD, n = 3 for *TOP3-AID* and *MSH4-AID TOP3-AID*; n = 6 for control and *MSH4-AID*). Statistical comparisons with control unless stated: ****P < 0.0001, ***P = 0.0005 (*TOP3-AID MSH4-AID* vs. control crossovers), ***P = 0.0001 (*TOP3-AID MSH4-AID* vs *MSH4-AID* crossovers), NS not significant *P* > 0.9999 (*TOP3-AID* vs. control) crossovers, *P* = 0.1250 (*TOP3-AID* vs. control non-crossovers), *P* = 0.0988 (*TOP3-AID MSH4-AID* vs. control non-crossovers), Tukey’s multiple comparisons test.

The stability of dHJs in pachytene-arrested cells when Rec8-AID and Top3-AID were degraded together indicated that resolution was again dependent on Plk1 and suggests that the crossover defect resulting from Rec8-AID degradation may be rescued. Indeed, expression of *NDT80-IN* while simultaneously degrading Rec8-AID and Top3-AID resulted in a 2.6-fold increase in crossovers relative to the degradation of Rec8-AID alone, whereas non-crossovers decreased by 1.6-fold (Fig. 5f–h). Note that the degradation of both Rec8-AID and Sgs1-AID gave similar results (Extended Data Fig. 7). Moreover, stabilization of dHJs and partial rescue of the crossover defects resulting from Zip1-AID or Msh4-AID degradation were also seen when Top3-AID was simultaneously degraded (Extended Data Figs. 8 and 9). Partial restoration of crossover levels is probably explained by our observation that when STR function was ablated, essentially all resolution was mediated by the Smc5/6–Mus81–Mms4 pathway (Extended Data Fig. 3).

**Extended Data Fig. 10:**
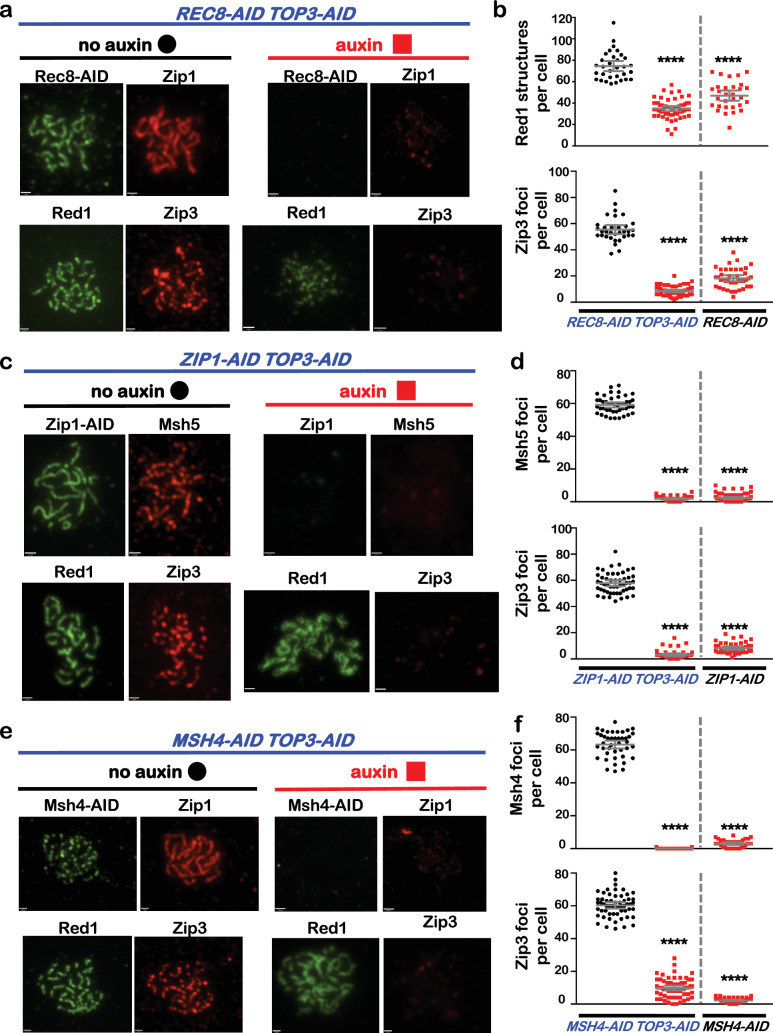
Stabilizing dHJs in pachytene does not bypass interdependencies between cohesin, synapsis, and crossover recombination complexes. **a**, Representative images of surface-spread meiotic nuclei from pachytene-arrested *REC8-AID TOP3-AID* cells sampled 1 hr after the addition of auxin or DMSO vehicle at 7 h, and immunostained for the indicated markers. **b**, Quantification of Red1 and Zip3 immunostaining structures from the *REC8-AID TOP3-AID* experiments represented in panel **a**, and comparison with corresponding data from *REC8-AID* (from Fig. 3b). Means ± SD for Red1 n = 32, 57, and 30 nuclei; and Zip3 n = 33, 57, and 42 nuclei. **c**, Representative images of surface-spread meiotic nuclei from pachytene-arrested *ZIP1-AID TOP3-AID* cells sampled 1 hr after the addition of auxin or DMSO vehicle at 7 h, and immunostained for the indicated markers. **d**, Quantification of Msh5 and Zip3 immunostaining foci from the experiments represented in panel **c**, and comparison with corresponding data from *ZIP1-AID* (from Fig. 4f). Means ± SD for Msh5 n = 44, 47, and 67 nuclei; and Zip3 n = 51, 44, and 41 nuclei. **e**, Representative images of surface-spread meiotic nuclei from pachytene-arrested *MSH4-AID TOP3-AID* cells sampled 1 hr after the addition of auxin or DMSO vehicle at 7 h, and immunostained for the indicated markers. **f**, Quantification of Msh4 and Zip3 immunostaining foci from the experiments represented in panel **e**, and comparison with corresponding data from *MSH4-AID* (from Fig. 4h). Means ± SD for Msh4 n = 42, 35, and 57 nuclei; and Zip3 n = 57, 63, and 49 nuclei. Scale bars = 1 μm. Unpaired two-tailed *t* test, ****P < 0.0001.

We wondered whether stabilizing dHJs also rescues the cytological defects that result from the degradation of Rec8-AID, Zip1-AID or Msh4-AID. To this end, Top3-AID was degraded together with Rec8-AID, Zip1-AID or Msh4-AID in pachytene-arrested cells, and chromosomes were immunostained for markers of synapsis (Zip1) and CRCs (Msh4 and Zip3) (Extended Data Fig. 10). In each case, cytological phenotypes were largely indistinguishable from those observed when Rec8-AID, Zip1-AID or Msh4-AID were degraded alone. SCs and CRCs disassembled after the degradation of both Rec8-AID and Top3-AID (Extended Data Fig. 10a,b), and CRCs were dissociated when Zip1-AID and Top3-AID were degraded together (Extended Data Fig. 10c,d). Moreover, SCs disassembled after the degradation of both Msh4-AID and Top3-AID (Extended Data Fig. 10e,f). Thus, the maintenance of inter-homologue DNA connections through the stabilization of dHJs does not bypass the interdependencies among cohesin, SCs and CRCs.

We conclude that the key components of pachytene chromosomes—cohesin-based homologue axes, SC transverse filaments and CRCs—protect crossover-designated dHJs from being aberrantly dissolved into non-crossovers by the STR complex (Extended Data Fig. 1b).

## Full synapsis is dispensable for crossovers

**Fig. 6:**
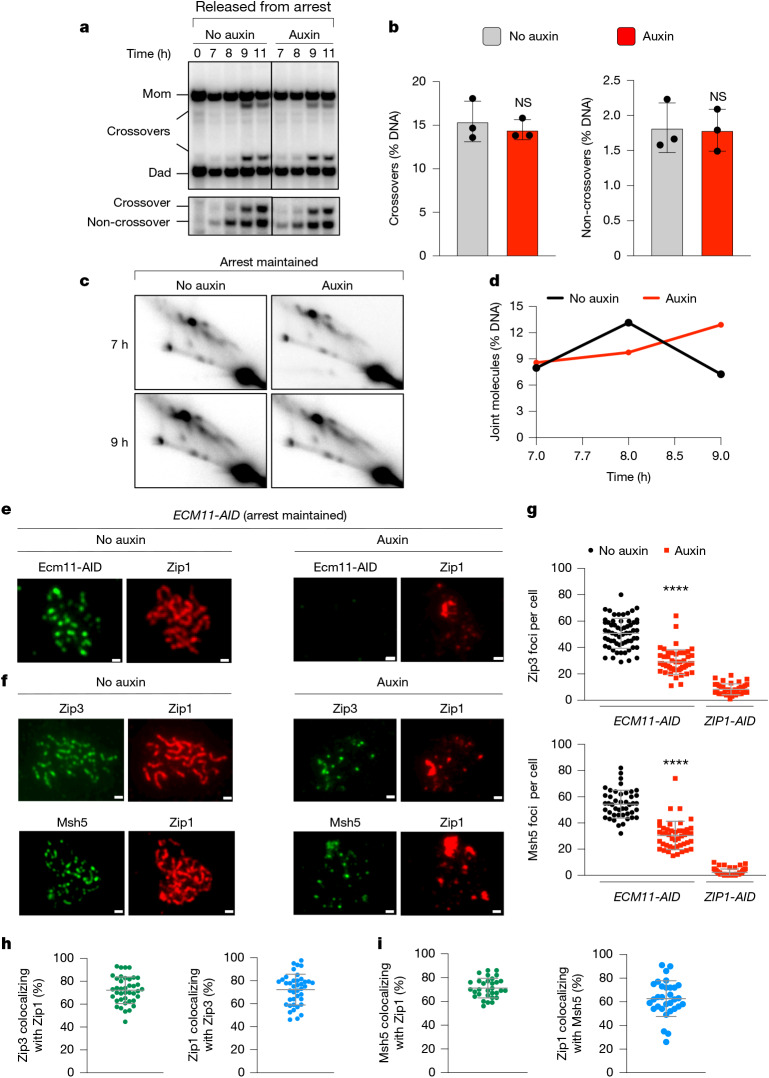
Full synapsis is not essential for crossover-specific dHJ resolution. **a**, Representative 1D gel Southern blot analysis of crossover (top) and non-crossover (bottom) formation from *ECM11-AID* subcultures (released from arrest) with or without auxin. **b**, Final levels of crossovers and non-crossovers at 11 h from the experiments in **a**. Mean ± s.d. of three independent experiments. NS, *P* > 0.9999, two-tailed, Mann–Whitney *U*-test. **c**, Representative 2D gel Southern blot analysis of joint molecules from *ECM11-AID* subcultures with and without the addition of auxin and pachytene arrest was maintained. **d**, Quantification of total joint molecule levels from the experiment in **c**. **e**,**f**, Representative images of surface-spread meiotic nuclei from pachytene-arrested *ECM11-AID* cells sampled 1 h after the addition of auxin or DMSO vehicle at 7 h and immunostained for the indicated markers. Scale bars, 1 μm. **g**, Quantification of foci stained for Zip3 and Msh5 from the *ECM11-AID* experiments in **e** and **f**, and comparison with corresponding data from *ZIP1-AID* (Fig. 4f). Mean ± s.d. Zip3 staining, *n* = 61, 51 and 41 nuclei; Msh5 staining, *n* = 45, 45 and 62 nuclei (*n* indicates the number of nuclei analysed for the auxin, no-auxin and *ZIP1-AID* experiments, respectively). Unpaired two-tailed *t*-test, ****P < 0.0001. **h**,**i**, Quantification of colocalization (mean ± s.d.) between foci immunostained for Zip1 and Zip3 (**h**, *n* = 40 nuclei), and Zip1 and Msh5 (**i**, *n* = 31 nuclei) from auxin-treated *ECM11-AID* nuclei in **e** and **f**.

High levels of crossing over can occur without end-to-end homologue synapsis in cells that lack the SC central element, which is required to mature and extend the transverse filament lattice to achieve full synapsis^5,6,10,40^. To determine whether the maintenance of full synapsis is required for crossover-specific dHJ resolution, the central element protein Ecm11 (ref. ^10^) was ablated as cells were released from pachytene arrest (Fig. 6). Crossover and non-crossover levels were indistinguishable from no-auxin controls when Ecm11-AID was degraded (Fig. 6a,b). This result was in marked contrast to the phenotypes seen after the loss of Rec8, Zip1 or Msh4 (Figs. 1 and 4). Moreover, in arrested *NDT80-IN* cells, dHJs remained stable after Ecm11-AID degradation (Fig. 6c,d).

Immunostaining revealed that SCs disassembled after Ecm11-AID degradation in arrested *NDT80-IN* cells, although numerous foci of Zip1 persisted (Fig. 6e). Thus, the central element is required to both establish and maintain full synapsis. Msh5 and Zip3 foci were maintained at about 50% of no-auxin control levels (Fig. 6f,g), which was in contrast to the almost complete loss seen after the degradation of Rec8-AID, Zip1-AID or Msh4-AID. Moreover, the remaining Msh5 and Zip3 foci showed high degrees of colocalization with residual Zip1 structures (Fig. 6h,i). These observations suggest that although mature SCs may stabilize a subset of CRCs, local ensembles of Zip1 and CRCs are sufficient to protect dHJs and support crossover-specific resolution.

## Discussion

The SC is an ancient structure that evolved with the emergence of sexual reproduction. Several functions are attributed to the SC. The assembly of homologue axes and pairwise synapsis of homologues establishes topological order in the nucleus. Once assembled, the SC also prevents the formation of DSBs and facilitates their repair to attenuate DNA-damage checkpoint signalling. At designated crossover sites, SC components facilitate dHJ formation^5^. This study, together with a companion paper^41^, reveals that key SC components, but not necessarily end-to-end synapsis per se, also enable crossover-specific resolution of dHJs by protecting them from unscheduled dissolution into non-crossovers by the conserved STR/BLM complex (Extended Data Fig. 1b,c). Failure of this crucial final step of meiotic recombination can result in unlinked univalent chromosomes that are prone to missegregation, which results in aneuploid gametes that are associated with infertility, miscarriage and congenital disease in humans.

Our data indicate that dHJs formed at crossover-designated recombination sites do not possess an intrinsic structure or topology that constrains their resolution fate to ensure crossing over. We previously proposed a model of crossover-specific resolution whereby asymmetric loading of PCNA during DNA synthesis associated with dHJ formation subsequently directs strand-specific nicking by the endonuclease MutLγ on both sides of the two Holliday junctions but distal to the exchange points^4^. This pattern of incisions always specifies a crossover outcome when the nicked dHJs are disassembled by the STR/BLM complex. Our two-step resolution model reconciles the counterintuitive pro-crossover role of STR/BLM during meiosis, which is in contrast to its well-characterized anticrossover roles in unwinding D-loops and dissolving unnicked dHJs into non-crossover products (Extended Data Figs. 1b and 3b,c). In this framework, we propose that mature dHJs are unnicked and therefore vulnerable to STR/BLM-catalysed dissolution until MutLγ is activated through the Ndt80-dependent expression of Plk1. By preventing STR/BLM from acting on dHJs until they have been nicked by MutLγ, SC components impose the sequential steps required for crossover-specific resolution (Extended Data Fig. 1b).

We suggest that local ensembles of cohesin and SC transverse filaments can be sufficient to protect dHJs by stabilizing CRCs, components of which can directly bind Holliday junctions, including MutSγ, MutLγ, Zip2–Spo16 (known as SHOC1–SPO16 in mammals) and Mer3 (known as HFM1 in mammals)^25,42^, and may directly compete with STR/BLM for binding dHJs and/or may constrain its activity. This proposal is consistent with electron microscopy visualization of late recombination nodules associated with short patches of SC in *Sordaria macrospora*^43^ and with the inference that in *C.* *elegans*, the cohesin local to designated crossover sites is distinctly regulated^28^. Moreover, components of the SC central region help recruit and stabilize CRCs^32,44–47^. Also in *C.* *elegans*, SC central region proteins seem to assemble transient crossover-specific compartments or ‘bubbles’ that may protect CRCs until crossover-specific resolution is executed^45^.

Our study, together with a companion paper^41^, also found that CRCs are required to maintain synapsis during pachytene. This observation is consistent with studies indicating that the SC central region is initially dynamic and labile but becomes more stable, contingent on the development of CRCs^48–51^. Notably, new subunits are continually incorporated into the SC central region after synapsis^48,51^. Moreover, in budding yeast, incorporation of new Zip1 molecules occurs predominantly at CRC sites^48^. Given that Zip1 seems to directly recruit CRC components^46,52^, this suggests a possible mechanism for the mutual stabilization of SCs and CRCs. This relationship may result in SCs with non-uniform structure and stability^48^, which could influence resolution fates and explain why sites of crossing over are the last to desynapse during diplotene^53^. The interdependence and dynamic nature of SCs and CRCs will render chromosomal interactions readily reversible until dHJs are resolved into crossovers. These attributes could help adjust and proofread homologous synapsis, minimize and resolve synaptic interlocks^54^ and preserve genome stability by destabilizing interactions between non-allelic and diverged sequences.

Importantly, dependency between synapsis and CRCs probably helps impose the ordered sequence of events required to ensure crossing over between homologues. At designated crossover sites, CRCs promote and maintain homologue synapsis, facilitate the formation of dHJs and protect them from aberrant dissolution. In turn, synapsis globally attenuates DSB formation^55^, thereby diminishing the DNA-damage kinase signalling that inhibits Ndt80 activity. Ndt80-dependent expression of Plk1 then activates crossover-specific dHJ resolution, CRCs disassemble and desynapsis ensues (Fig. 1c,d).

Our results also revealed that two SMC complexes mediate essentially all Plk1-dependent joint-molecule resolution during meiosis (Extended Data Fig. 1c). Rec8–cohesin is required to maintain synapsis and CRCs and thereby protects dHJs to facilitate their crossover-specific resolution. Whether local or global functions of Rec8–cohesin are important for crossover-specific dHJ resolution, and the roles of cohesive versus chromatin-loop-organizing populations of Rec8–cohesin, remain unclear. A distinct Smc5/6-dependent pathway is essential for resolution through Mus81–Mms4 and a subpopulation of STR/BLM, producing a mixture of crossovers and non-crossovers. Despite these distinctions, Rec8–cohesin and Smc5/6 could have common functions to constrain favourable joint-molecule topology^56^ and control access by resolving enzymes.

## Methods

### Data reporting

No statistical methods were used to predetermine sample sizes. The experiments were not randomized, and the investigators were not blinded to allocation during experiments. Blinding was used during outcome assessment for the cytology experiments.

### Yeast strains

For full genotypes, see Supplementary Table 1. The AID system^11^ was optimized for meiosis by replacing the promoter of the *pADH1-OsTIR1* cassette with the *CUP1* promoter^36^. Carboxy-terminal fusion of a minimal AID to targeted proteins was constructed using the plasmid pHyg-AID*−9Myc as the template for PCR-mediated allele replacement^57^. To construct an internal degron allele of *ZIP1*, AID sequences were inserted into plasmid pMPY-3×HA and integrated after codon 700 through PCR epitope tagging^58^. The primers used to construct AID alleles are listed in Supplementary Table 2. The oestrogen-inducible *NDT80-IN* *GAL4-ER* system has been previously described^59–61^.

### Meiotic time courses and DNA physical assays

Detailed protocols for meiotic time courses and DNA physical assays at the *HIS4::LEU2* locus have been previously described^62^. At 6.5 h after the induction of meiosis, CuSO_4_ (100 mM stock in dH_2_O) was added to obtain a final concentration of 50 μM to induce the expression of *pCUP1-OsTIR1* (which encodes the E3 ligase Tir1) and cell cultures were split. For experiments in which *NDT80-IN* was induced, oestradiol (5 mM stock, Sigma E2758 (β-estradiol) in ethanol) was added at 7 h to obtain a final concentration of 1 μM to both subcultures. Simultaneously, auxin (3-indoleacetic acid, Sigma, 13750, 2 M stock in DMSO) was added to one subculture to obtain a final concentration of 2 mM; an equivalent volume of DMSO was added to the no-auxin control subculture. At 7.5 h, auxin was added again at 1 mM. To analyse the timing and efficiency of meiotic divisions and sporulation, cells were fixed in 40% ethanol, 0.1 M sorbitol, stained with DAPI, and around 200 cells were categorized for each time point. For imaging, DAPI-stained cells were mounted in antifade (Vectashield, Vector Laboratories) and digital images were captured using a Zeiss Axioplan II microscope, Hamamatsu ORCA-ER CCD camera and Volocity software.

### Chromosome spreading and immunofluorescence microscopy

Cell samples were collected and processed for chromosome spreading and immunostaining essentially as previously described^63^. The following primary antibodies were provided by A. Shinohara: chicken anti-Red1 (1:500 dilution); rabbit anti-Msh5 (1:750); and rabbit anti-Zip3 (1:500). Guinea pig anti-Zip1 (1:400) was a gift from S. Keeney. Monoclonal anti-cMyc (1:1,000, Roche 11667149001) was used to detect AID–9myc fused proteins. All primary antibodies were incubated overnight at room temperature in 100 μl TBS–BSA buffer (10 mM Tris pH 7.5, 150 mM NaCl and 1% BSA). The secondary antibodies anti-rabbit 568 (A11036, Molecular Probes, 1:1,000), anti-mouse 488 (A11029, Molecular Probes, 1:1,000), anti-rabbit 647 (A21245, Invitrogen) and anti-guinea pig 555 (A21435, Life Technologies) were incubated for 1 h at 37 °C. Coverslips were mounted with Prolong Gold antifade reagent (Invitrogen, P36930). Digital images were captured using a Zeiss Axioplan II microscope, Hamamatsu ORCA-ER CCD camera and analysed using Volocity software. Scatterplots were generated using the GraphPad program in Prism.

### Western blot analysis

Whole-cell extracts were prepared using a TCA extraction method essentially as previously described^64^. Samples were analysed by standard SDS–PAGE and western blotting using the following primary antibodies: monoclonal anti-cMyc (1:1,000, Roche, 11667149001); monoclonal anti-HA (1:1,000, Sigma, 11583816001); goat polyclonal Arp7 (1:10,000, Santa Cruz, SC-8960); goat polyclonal Zip1 (1:500, Santa Cruz, y-N16); rabbit anti-Msh4 (1:500); rabbit anti-Msh5 (1:500); and rabbit anti-Zip3 (1:500; Msh4, Msh5 and Zip3 antibodies were a gift from A. Shinohara). The secondary antibodies (1:5,000) were IRDye 800CW donkey anti-mouse IgG (LI-COR, 925-32212), IRDye 680LT donkey anti-goat IgG (LI-COR, 925-68024), IRDye 680LT donkey anti-rabbit IgG (LI-COR, 925-68023) and IRDye 800CW donkey anti-rabbit IgG (LI-COR, 925-32213). Western blots were imaged on an Odyssey Infrared Imager (LI-COR), and quantification of protein bands was performed using Image Studio Lite (v.5.0.021) software.

### Statistical analysis and reproducibility

Statistical analyses were performed using Prism 8 (GraphPad Software). For bar graphs and scatter plots comparing two samples (aggregated from three or more replicate experiments), unpaired *t*-tests were performed. For multiple comparisons, one-way analysis of variance was performed (Tukey’s or Dunnett’s tests were used depending on the specific comparisons being made). For scatter plots and bar graphs, error bars show the mean value with standard deviations. Western blots are representative of at least three repeats (Figs. 1b, 4ab and 5a,d and Extended Data Figs. 2a, 3a,d,e, 4a,b, 6a,d, 8a,d and 9a,d). Representative immunofluorescence images were chosen from more than 30 samples analysed from at least three biological replicates (Fig. 3a, 4e,g and 6e,g and Extended Data Fig. 10a,c,e).

### Reporting summary

Further information on research design is available in the Nature Portfolio Reporting Summary linked to this article.

## Online content

Any methods, additional references, Nature Portfolio reporting summaries, source data, extended data, supplementary information, acknowledgements, peer review information; details of author contributions and competing interests; and statements of data and code availability are available at 10.1038/s41586-025-09555-1.

## Supplementary information

Supplementary Fig. 1 Source gel images with cropping indicated by red or white boxes.

Reporting Summary

Supplementary Tables Supplementary Table 1. *Saccharomyces cerevisiae* strains used in this study. Supplementary Table 2. Oligonucleotides used in this study.

## Data Availability

Relevant data generated or analysed during this study are included in this Article and its Supplementary Information files. Biological materials are available from the corresponding author.
