# Supplementary material for: Protecting double Holliday junctions ensures crossing over during meiosis
Source: Nature. 2025 Sep 24;647(8090):776–85. doi: 10.1038/s41586-025-09555-1 (PMC12629981; doi:10.1038/s41586-025-09555-1)
Supplement: Supplementary file 2 — Reporting Summary [file 41586_2025_9555_MOESM2_ESM.pdf]

nature portfolio

Corresponding author(s): Neil Hunter

Last updated by author(s): 07/25/2025

Reporting Summary

Nature Portfolio wishes to improve the reproducibility of the work that we publish. This form provides structure for consistency and transparency in reporting. For further information on Nature Portfolio policies, see our Editorial Policies and the Editorial Policy Checklist.

Statistics

For all statistical analyses, confirm that the following items are present in the figure legend, table legend, main text, or Methods section.

n/a	Confirmed	
☐	☒	The exact sample size (*n*) for each experimental group/condition, given as a discrete number and unit of measurement
☐	☒	A statement on whether measurements were taken from distinct samples or whether the same sample was measured repeatedly
☐	☒	The statistical test(s) used AND whether they are one- or two-sided<br>*Only common tests should be described solely by name; describe more complex techniques in the Methods section.*
☒	☐	A description of all covariates tested
☐	☒	A description of any assumptions or corrections, such as tests of normality and adjustment for multiple comparisons
☐	☒	A full description of the statistical parameters including central tendency (e.g. means) or other basic estimates (e.g. regression coefficient) AND variation (e.g. standard deviation) or associated estimates of uncertainty (e.g. confidence intervals)
☐	☒	For null hypothesis testing, the test statistic (e.g. F, t, r) with confidence intervals, effect sizes, degrees of freedom and P value noted<br>*Give P values as exact values whenever suitable.*
☒	☐	For Bayesian analysis, information on the choice of priors and Markov chain Monte Carlo settings
☒	☐	For hierarchical and complex designs, identification of the appropriate level for tests and full reporting of outcomes
☒	☐	Estimates of effect sizes (e.g. Cohen's *d*, Pearson's *r*), indicating how they were calculated

Our web collection on statistics for biologists contains articles on many of the points above.

Software and code

Policy information about availability of computer code

Data collection	Fluro Chem 8900 (alpha innotech), Typhoon FLA 9500, GE Healthcare, odyssey Imager (LI-COR), Zeiss Axioplan II microscope, Hamamatsu ORCA-ER CCD camera.
Data analysis	Fiji Image J 1.45s, Graphpad Prism 8, ImageQuant 7.0 (GE Healthcare), Image Studio Lite 5.0.21, Volocity 6.1.1 (Perkin Elmer)

For manuscripts utilizing custom algorithms or software that are central to the research but not yet described in published literature, software must be made available to editors and reviewers. We strongly encourage code deposition in a community repository (e.g. GitHub). See the Nature Portfolio guidelines for submitting code & software for further information.

Data

Policy information about availability of data

All manuscripts must include a data availability statement. This statement should provide the following information, where applicable:

- Accession codes, unique identifiers, or web links for publicly available datasets
- A description of any restrictions on data availability
- For clinical datasets or third party data, please ensure that the statement adheres to our policy

Relevant data generated or analyzed during this study are included in this Article and its Supplementary Information files. All the uncropped images are available as a single .xls file, "Supplemental Figure 1". Biological materials are available from the corresponding author.

Research involving human participants, their data, or biological material

Policy information about studies with human participants or human data. See also policy information about sex, gender (identity/presentation), and sexual orientation and race, ethnicity and racism.

Reporting on sex and gender	N/A
Reporting on race, ethnicity, or other socially relevant groupings	N/A
Population characteristics	N/A
Recruitment	N/A
Ethics oversight	N/A

Note that full information on the approval of the study protocol must also be provided in the manuscript.

Field-specific reporting

Please select the one below that is the best fit for your research. If you are not sure, read the appropriate sections before making your selection.

☒ Life sciences ☐ Behavioural & social sciences ☐ Ecological, evolutionary & environmental sciences

For a reference copy of the document with all sections, see nature.com/documents/nr-reporting-summary-flat.pdf

Life sciences study design

All studies must disclose on these points even when the disclosure is negative.

Sample size	Preliminary experiments were performed to determine sample sizes. Sample size was determined to be adequate based on the magnitude and consistency of measurable differences between groups. Statistical methods to predetermine sample-size were not performed. All sample sizes are reported in the Results, Legends or Methods sections.
Data exclusions	No data were excluded.
Replication	No attempts for replication failed.The numbers of all replicates for each experiment are indicated in the figure legends.
Randomization	The cells quantified in each experiment were randomly sampled from the total population of cells.
Blinding	During cytology experiments, samples were assigned a number and the investigator was blinded to the numbering. Blinding was not applicable for other experiments due to lack of randomizable treatments/interventions in the experimental plan

Reporting for specific materials, systems and methods

We require information from authors about some types of materials, experimental systems and methods used in many studies. Here, indicate whether each material, system or method listed is relevant to your study. If you are not sure if a list item applies to your research, read the appropriate section before selecting a response.

Materials & experimental systems

n/a	Involved in the study	
☐	☒	Antibodies
☒	☐	Eukaryotic cell lines
☒	☐	Palaeontology and archaeology
☒	☐	Animals and other organisms
☒	☐	Clinical data
☒	☐	Dual use research of concern
☒	☐	Plants

Methods

n/a	Involved in the study	
☒	☐	ChIP-seq
☒	☐	Flow cytometry
☒	☐	MRI-based neuroimaging

Antibodies

Antibodies used	For cytology, primary antibodies were chicken anti-Red1 (1:500 dilution, a gift from Akira Shinohara), rabbit anti-Msh5 (1:750, a gift from Akira Shinohara), and rabbit anti-Zip3 (1:500, a gift from Akira Shinohara); guinea pig anti-Zip1 (1:400, a gift from Scott Keeney);

moncolonal anti-c-Myc (1:1000, Roche 11667149001) was used to detect AID-9myc fused proteins. Secondary antibodies were anti-rabbit 568 (A11036 Molecular Probes, 1:1,000), anti-mouse 488 (A11029 Molecular Probes, 1:1,000), anti-rabbit 647 (A21245 Invitrogen), and anti-guinea pig 555 (A21435 Life Technologies).
For Western blots. primary antibodies were monoclonal anti-c-Myc (1:1000, Roche 11667149001), monoclonal anti-HA (1:1000, Sigma 11583816001), goat anti-Arp7 (1:10,000, Santa Cruz SC-8960), rabbit anti-Msh4 (1:500, a gift from Akira Shinohara), rabbit anti-Msh5 (1:500 a gift from Akira Shinohara), and guinea pig anti-Zip1 (1/500, . Secondary antibodies (1:5000) were IRDye® 800CW Donkey anti-Mouse IgG (LI-COR 925-32212), IRDye® 680LT Donkey anti-Goat IgG (LI-COR 925-68024), IRDye® 680LT Donkey anti-Rabbit IgG (LI-COR 925-68023) and IRDye® 800CW Donkey anti-Rabbit IgG (LI-COR 925-32213).

Validation

All commercial antibodies used in this study were certified and validated by manufacturers and vendors. Yeast Red1, Msh4, Msh5, Zip1, and Zip3 antibodies were previously validated in several published studies (citations below). Validation of c-Myc, HA, and Arp7 antibodies are described at the manufacturers websites (below). Specificities of all primary antibodies were also confirmed in our lab using appropriate negative controls (deletion mutants and untagged alleles).

Shinohara M, Shinohara A. The Msh5 complex shows homeostatic localization in response to DNA double-strand breaks in yeast meiosis. Front Cell Dev Biol. 2023 May 18;11:1170689. doi: 10.3389/fcell.2023.1170689. PMID: 37274743; PMCID: PMC10232913.
Shinohara M, Oh SD, Hunter N, Shinohara A. Crossover assurance and crossover interference are distinctly regulated by the ZMM proteins during yeast meiosis. Nat Genet. 2008 Mar;40(3):299-309. doi: 10.1038/ng.83. Epub 2008 Feb 24. PMID: 18297071.
Li K, Yoshimura K, Shinohara M. Meiotic DNA double-strand break-independent role of protein phosphatase 4 in Hop1 assembly to promote meiotic chromosome axis formation in budding yeast. Genes Cells. 2023 Aug;28(8):595-614. doi: 10.1111/gtc.13049. Epub 2023 May 27. PMID: 37243502.
Zhu Z, Mori S, Oshiumi H, Matsuzaki K, Shinohara M, Shinohara A. Cyclin-dependent kinase promotes formation of the synaptonemal complex in yeast meiosis. Genes Cells. 2010 Oct;15(10):1036-50. doi: 10.1111/j.1365-2443.2010.01440.x. Epub 2010 Sep 6. PMID: 20825495.
Sasanuma H, Tawaramoto MS, Lao JP, Hosaka H, Sanda E, Suzuki M, Yamashita E, Hunter N, Shinohara M, Nakagawa A, Shinohara A. A new protein complex promoting the assembly of Rad51 filaments. Nat Commun. 2013;4:1676. doi: 10.1038/ncomms2678. PMID: 23575680; PMCID: PMC4353811.
Arora C, Kee K, Maleki S, Keeney S. Antiviral protein Ski8 is a direct partner of Spo11 in meiotic DNA break formation, independent of its cytoplasmic role in RNA metabolism. Mol Cell. 2004 Feb 27;13(4):549-59. doi: 10.1016/s1097-2765(04)00063-2. PMID: 14992724.
Bhagwat NR, Owens SN, Ito M, Boinapalli JV, Poa P, Ditzel A, Kopparapu S, Mahalawat M, Davies OR, Collins SR, Johnson JR, Krogan NJ, Hunter N. SUMO is a pervasive regulator of meiosis. Elife. 2021 Jan 27;10:e57720. doi: 10.7554/eLife.57720. PMID: 33502312; PMCID: PMC7924959.
Goat anti-Zip1 (discontinued): https://datasheets.scbt.com/sc-15632.pdf
c-myc: https://www.sigmaaldrich.com/US/en/product/roche/roamyc
HA: https://www.sigmaaldrich.com/US/en/product/roche/roaha?srsltid=AfmBOoqJ5HdM6ETik9R6VqzzcviJwTIinpqXAG8bumW-MG_LIsByeMWW
Arp7 (discontinued) https://www.citeab.com/antibodies/3430430-sc-8961-arp7-yc-20

Plants

Seed stocks

N/A

Novel plant genotypes

N/A

Authentication

N/A
