# Supplementary material for: Protecting double Holliday junctions ensures crossing over during meiosis
Source: Nature. 2025 Sep 24;647(8090):776–85. doi: 10.1038/s41586-025-09555-1 (PMC12629981; doi:10.1038/s41586-025-09555-1)
Supplement: Supplementary file 3 — Supplementary Table 1. Saccharomyces cerevisiae strains used in this study. Supplementary Table 2. Oligonucleotides used in this study. [file 41586_2025_9555_MOESM3_ESM.pdf]

**Supplementary Table 1.** ***Saccharomyces cerevisiae*** **strains used in this study**	
Strain	Genotype
NHY 7291	*MATa/MATα HIS4::LEU2-(BamHI)/his4-X::LEU2-(NgoMIV)—URA3 TOP3-AID-9myc::hphMX4 NSE4-AID-9myc::hphMX4 /TOP3-AID-9myc::hphMX4 NSE4-AID-9myc::hphMX4 hphMX4::PGAL1-NDT80/hphMX4::PGAL1-NDT80 ura3:PGPD1-GAL4(848)-ER:URA3 hphMX4::PCUP1-1-OsTIR1::lys2/ PCUP1-1-OsTIR1-9Myc-URA3*
NHY7475	*MATa/MATα HIS4::LEU2-(BamHI)/his4-X::LEU2-(NgoMIV)—URA3 NSE4-AID-9myc::hphMX4 /NSE4-AID-9myc::hphMX4 hphMX4::PGAL1-NDT80/hphMX4::PGAL1-NDT80 ura3:PGPD1-GAL4(848)-ER:URA3 hphMX4::PCUP1-1-OsTIR1::lys2/ PCUP1-1-OsTIR1-9Myc-URA3*
NHY7699	*MATa/MATα HIS4::LEU2-(BamHI)/his4-X::LEU2-(NgoMIV)—URA3 REC8-AID-9myc::hphMX4 NSE4-AID-9myc::hphMX4 /REC8-AID-9myc::hphMX4 NSE4-AID-9myc::hphMX4 hphMX4::PGAL1-NDT80/hphMX4::PGAL1-NDT80 ura3:PGPD1-GAL4(848)-ER:URA3 hphMX4::PCUP1-1-OsTIR1::lys2/ PCUP1-1-OsTIR1-9Myc-URA3*
NHY7824	*MATa/MATα HIS4::LEU2-(BamHI)/his4-X::LEU2-(NgoMIV)—URA3 REC8-AID-9myc::hphMX4 /REC8-AID-9myc::hphMX4 hphMX4::PGAL1-NDT80/hphMX4::PGAL1-NDT80 ura3:PGPD1-GAL4(848)-ER:URA3 hphMX4::PCUP1-1-OsTIR1::lys2/ PCUP1-1-OsTIR1-9Myc-URA3*
NHY7854	*MATa/MATα HIS4::LEU2-(BamHI)/his4-X::LEU2-(NgoMIV)—URA3 NSE4-AID-9myc::hphMX4 csm2Δ::KanMX6 /NSE4-AID-9myc::hphMX4 csm2Δ::KanMX6 hphMX4::PGAL1-NDT80/hphMX4::PGAL1-NDT80 ura3:PGPD1-GAL4(848)-ER:URA3 hphMX4::PCUP1-1-OsTIR1::lys2/ PCUP1-1-OsTIR1-9Myc-URA3*
NHY7914	*MATa/MATα HIS4::LEU2-(BamHI)/his4-X::LEU2-(NgoMIV)—URA3 NSE4-AID-9myc::hphMX4 mph1::KanMX6/NSE4-AID-9myc::hphMX4 mph1::KanMX6 hphMX4::PGAL1-NDT80/hphMX4::PGAL1-NDT80 ura3:PGPD1-GAL4(848)-ER:URA3 hphMX4::PCUP1-1-OsTIR1::lys2/ PCUP1-1-OsTIR1-9Myc-URA3*
NHY7988	*MATa/MATα HIS4::LEU2-(BamHI)/his4-X::LEU2-(NgoMIV)—URA3 REC8-AID-9myc::hphMX4 mlh3Δ::kanMX4 /REC8-AID-9myc::hphMX4 mlhΔ::kanMX4 hphMX4::PGAL1-NDT80/hphMX4::PGAL1-NDT80 ura3:PGPD1-GAL4(848)-ER:URA3 hphMX4::PCUP1-1-OsTIR1::lys2/ PCUP1-1-OsTIR1-9Myc-URA3*
NHY8111	*MATa/MATα HIS4::LEU2-(BamHI)/his4-X::LEU2-(NgoMIV)—URA3 REC8-AID-9myc::hphMX4 TOP3-AID-9myc::hphMX4/REC8-AID-9myc::hphMX4 TOP3-AID-9myc::hphMX4 hphMX4::PGAL1-NDT80/hphMX4::PGAL1-NDT80 ura3:PGPD1-GAL4(848)-ER:URA3 hphMX4::PCUP1-1-OsTIR1::lys2/ PCUP1-1-OsTIR1-9Myc-URA3*
NHY8177	*MATa/MATα HIS4::LEU2-(BamHI)/his4-X::LEU2-(NgoMIV)—URA3 TOP3-AID-9myc::hphMX4 /TOP3-AID-9myc::hphMX4 hphMX4::PGAL1-NDT80/hphMX4::PGAL1-NDT80 ura3:PGPD1-GAL4(848)-ER:URA3 hphMX4::PCUP1-1-OsTIR1::lys2/ PCUP1-1-OsTIR1-9Myc-URA3*
NHY8256	*MATa/MATα HIS4::LEU2-(BamHI)/his4-X::LEU2-(NgoMIV)—URA3 SMC3-AID-9myc::hphMX4 /SMC3-AID-9myc::hphMX4 hphMX4::PGAL1-NDT80/hphMX4::PGAL1-NDT80 ura3:PGPD1-GAL4(848)-ER:URA3 hphMX4::PCUP1-1-OsTIR1::lys2/ PCUP1-1-OsTIR1-9Myc-URA3*
NHY8263	*MATa/MATα HIS4::LEU2-(BamHI)/his4-X::LEU2-(NgoMIV)—URA3 SMC3-AID-9myc::hphMX4 NSE4-AID-9myc::hphMX4 /SMC3-AID-9myc::hphMX4 NSE4-AID-9myc::hphMX4 hphMX4::PGAL1-NDT80/hphMX4::PGAL1-NDT80 ura3:PGPD1-GAL4(848)-ER:URA3 hphMX4::PCUP1-1-OsTIR1::lys2/ PCUP1-1-OsTIR1-9Myc-URA3*
NHY8500	*MATa/MATα HIS4::LEU2-(BamHI)/his4-X::LEU2-(NgoMIV)—URA3 SMC3-AID-9myc::hphMX4 TOP3-AID-9myc::hphMX4 /SMC3-AID-9myc::hphMX4 TOP3-AID-9myc::hphMX4 hphMX4::PGAL1-NDT80/hphMX4::PGAL1-NDT80 ura3:PGPD1-GAL4(848)-ER:URA3 hphMX4::PCUP1-1-OsTIR1::lys2/ PCUP1-1-OsTIR1-9Myc-URA3*
NHY8555	*MATa/MATα HIS4::LEU2-(BamHI)/his4-X::LEU2-(NgoMIV)—URA3 MMS4-AID-9myc::hphMX4 /MMS4-AID-9myc::hphMX4 hphMX4::PGAL1-NDT80/hphMX4::PGAL1-NDT80 ura3:PGPD1-GAL4(848)-ER:URA3 hphMX4::PCUP1-1-OsTIR1::lys2/ PCUP1-1-OsTIR1-9Myc-URA3*
NHY8714	*MATa/MATα HIS4::LEU2-(BamHI)/his4-X::LEU2-(NgoMIV)—URA3 SMC3-AID-9myc::hphMX4 MMS4-AID-9myc::hphMX4 yen1Δ::KanMX6 / SMC3-AID-9myc::hphMX4 MMS4-AID-*

	9myc::hphMX4 yen1Δ::KanMX6 hphMX4::PGAL1-NDT80/hphMX4::PGAL1-NDT80 ura3:PGPD1-GAL4(848)-ER:URA3 hphMX4::PCUP1-1-OsTIR1::lys2/ PCUP1-1-OsTIR1-9Myc-URA3
NHY8793	*MATa/MATα HIS4::LEU2-(BamHI)/his4-X::LEU2-(NgoMIV)—URA3 SGS1-AID-9myc::hphMX4 NSE4-AID-9myc::hphMX4 /SGS1-AID-9myc::hphMX4 NSE4-AID-9myc::hphMX4 hphMX4::PGAL1-NDT80/hphMX4::PGAL1-NDT80 ura3:PGPD1-GAL4(848)-ER:URA3 hphMX4::PCUP1-1-OsTIR1::lys2/ PCUP1-1-OsTIR1-9Myc-URA3*
NHY8806	*MATa/MATα HIS4::LEU2-(BamHI)/his4-X::LEU2-(NgoMIV)—URA3 SGS1-AID-9myc::hphMX4 /SGS1-AID-9myc::hphMX4 hphMX4::PGAL1-NDT80/hphMX4::PGAL1-NDT80 ura3:PGPD1-GAL4(848)-ER:URA3 hphMX4::PCUP1-1-OsTIR1::lys2/ PCUP1-1-OsTIR1-9Myc-URA3*
NHY8873	*MATa/MATα HIS4::LEU2-(BamHI)/his4-X::LEU2-(NgoMIV)—URA3 TOP3-AID-9myc::hphMX4 MMS4-AID-9myc::hphMX4 yen1Δ::KanMX6 /TOP3-AID-9myc::hphMX4 MMS4-AID-9myc::hphMX4 yen1Δ::KanMX6 hphMX4::PGAL1-NDT80/hphMX4::PGAL1-NDT80 ura3:PGPD1-GAL4(848)-ER:URA3 hphMX4::PCUP1-1-OsTIR1::lys2/ PCUP1-1-OsTIR1-9Myc-URA3*
NHY8875	*MATa/MATα HIS4::LEU2-(BamHI)/his4-X::LEU2-(NgoMIV)—URA3 MMS4-AID-9myc::hphMX4 yen1Δ::KanMX6 /MMS4-AID-9myc::hphMX4 yen1Δ::KanMX6 hphMX4::PGAL1-NDT80/hphMX4::PGAL1-NDT80 ura3:PGPD1-GAL4(848)-ER:URA3 hphMX4::PCUP1-1-OsTIR1::lys2/ PCUP1-1-OsTIR1-9Myc-URA3*
NHY8880	*MATa/MATα HIS4::LEU2-(BamHI)/his4-X::LEU2-(NgoMIV)—URA3 TOP3-AID-9myc::hphMX4 NSE4-AID-9myc::hphMX4 mph1::KanMX6 /TOP3-AID-9myc::hphMX4 NSE4-AID-9myc::hphMX4 mph1::KanMX6 hphMX4::PGAL1-NDT80/hphMX4::PGAL1-NDT80 ura3:PGPD1-GAL4(848)-ER:URA3 hphMX4::PCUP1-1-OsTIR1::lys2/ PCUP1-1-OsTIR1-9Myc-URA3*
NHY9078	*MATa/MATα HIS4::LEU2-(BamHI)/his4-X::LEU2-(NgoMIV)—URA3 ZIP1-AID-3HA /ZIP1-AID-3HA hphMX4::PGAL1-NDT80/hphMX4::PGAL1-NDT80 ura3:PGPD1-GAL4(848)-ER:URA3 hphMX4::PCUP1-1-OsTIR1::lys2/ PCUP1-1-OsTIR1-9Myc-URA3*
NHY9117	*MATa/MATα HIS4::LEU2-(BamHI)/his4-X::LEU2-(NgoMIV)—URA3 RMI1-AID-9myc::hphMX4 NSE4-AID-9myc::hphMX4 /RMI1-AID-9myc::hphMX4 NSE4-AID-9myc::hphMX4 hphMX4::PGAL1-NDT80/hphMX4::PGAL1-NDT80 ura3:PGPD1-GAL4(848)-ER:URA3 hphMX4::PCUP1-1-OsTIR1::lys2/ PCUP1-1-OsTIR1-9Myc-URA3*
NHY9263	*MATa/MATα HIS4::LEU2-(BamHI)/his4-X::LEU2-(NgoMIV)—URA3 ECM11-AID-9myc::hphMX4 /ECM11-AID-9myc::hphMX4 hphMX4::PGAL1-NDT80/hphMX4::PGAL1-NDT80 ura3:PGPD1-GAL4(848)-ER:URA3 hphMX4::PCUP1-1-OsTIR1::lys2/ PCUP1-1-OsTIR1-9Myc-URA3*
NHY9347	*MATa/MATα HIS4::LEU2-(BamHI)/his4-X::LEU2-(NgoMIV)—URA3 MSH4-AID-9myc::hphMX4 /MSH4-AID-9myc::hphMX4 hphMX4::PGAL1-NDT80/hphMX4::PGAL1-NDT80 ura3:PGPD1-GAL4(848)-ER:URA3 hphMX4::PCUP1-1-OsTIR1::lys2/ PCUP1-1-OsTIR1-9Myc-URA3*
NHY9525	*MATa/MATα HIS4::LEU2-(BamHI)/his4-X::LEU2-(NgoMIV)—URA3 TOP3-AID-9myc::hphMX4 ZIP1-AID-3HA /TOP3-AID-9myc::hphMX4 ZIP1-AID-3HA hphMX4::PGAL1-NDT80/hphMX4::PGAL1-NDT80 ura3:PGPD1-GAL4(848)-ER:URA3 hphMX4::PCUP1-1-OsTIR1::lys2/ PCUP1-1-OsTIR1-9Myc-URA3*
NHY9554	*MATa/MATα HIS4::LEU2-(BamHI)/his4-X::LEU2-(NgoMIV)—URA3 MSH4-AID-9myc::hphMX4 TOP3-AID-9myc::hphMX4/MSH4-AID-9myc::hphMX4 TOP3-AID-9myc::hphMX4 hphMX4::PGAL1-NDT80/hphMX4::PGAL1-NDT80 ura3:PGPD1-GAL4(848)-ER:URA3 hphMX4::PCUP1-1-OsTIR1::lys2/ PCUP1-1-OsTIR1-9Myc-URA3*
* In addition, all strains contain the markers *leu2::hisG, ura3(Δsma-pst)* and *ho::hisG*	

Supplementary Table 2. Oligonucleotides used in this study	
REC8-AID primer 1	TGCGGTAACTAAAGATCTTAAATTGAGAAGAGAGGACGAAATAATTGTATATGCCCGTACGCTGCAGGTCGAC
REC8-AID primer 2	GAGGACAGCGGCTAGTAACCGCTGTCCTCATATGGAAGGAGAAAATAAAAATTCAATCGATGAATTCGAGCTCG
NSE4-AID primer1	GGACCAGCTATGAAAAAAAAAAAAAAAAAAAAAAAAAAAACTGTACATATTATATGCAGCGCTCTATCGCTGTTAATCGATGAATTCGAGCTCG
NSE4-AID primer1	ATTATTTTTCAAATGGACATGCCTACTTGGCGAAAACTAATAAAGAAATACAACATCACTTCACCATTCTTAGACCGTACGCTGCAGGTCGAC
TOP3-AID primer1	GAATGCCTGCAAGAATACTCTCTTGCAAGTTTATGACCGTGTCAAGGCGTCCATGCGTACGCTGCAGGTCGAC
TOP3-AID primer2	TCATGCAATTAAGCGGAGGGCTTTTTCGAAGACAAAAGGCGGCAAAAACGCCTTAATCGATGAATTCGAGCTCG
MMS4-AID primer1	GAGGCAGTAGAAAAAAGATTGTACAAACTGTTTACTTGTACTGATCCAAATGATACTATTGAACGTACGCTGCAGGTCGAC
MMS4-AID Primer2	GCAGTGATTTTCAAAACGACTGCCTTAAGGTATGTTCTTATATACAAAGTTTCGTTCGATCATCAATCGATGAATTCGAGCTCG
SGS1-AID Primer1	TGCTAATGGGAGACGAGGTTTTAGAAATTACCGAGGTCACTATAGAGGAAGAAAGCGTACGCTGCAGGTCGAC
SGS1-AID Primer2	GCTTGGCGAATGGTGTCGTAGTTATAAGTAACACTATTTATTTTTCTACTCTTCAATCGATGAATTCGAGCTCG
ZIP1 internal AID Primer1	GAGGAATCACTAAGCGATGTAAAAACCCTAAAACAGCAAGTGATAGTTTTGAAATCGGAGAAGCAAGATATAACAAGGGAACAAAAGCTGGAG
ZIP1 interal primer2	GTAAATTTTTGGTGACTTCTTCCAAACTTTCGAGGTTATCTTGAAGTTCTAACTTTTCGGCGCCACCTCCGCCTCCACCCTGTAGGGCGAATTGGGTAC
SMC3-AID Primer1	GTTATTGAGGTCAATAGAGAAGAAGCAATCGGATTCATTAGAGGTAGCAATAAATTCGCTGAAGTCCGTACGCTGCAGGTCGAC
SMC3-AID Primer2	CAAATAGCTATTTATGTAAGCAAAACTGATATTTTTATATACAAACCGTTTCAAATATCTCTTAATCGATGAATTCGAGCTCG
MCD1-AID Primer1	GTCAAACAGAAGCATTCGGAAATATTAAAATAGACGCCAAACCTGCACTATTTGAAAGGTTTATCAATGCTCGTACGCTGCAGGTCGAC
MCD1-AID Primer2	GTCTTTGATCTATATATGCATCAGCTTACTGGGTCCACCAAGAAATCCCCTCGGCGTAACTAGGTTTTAATCGATGAATTCGAGCTCG
ESP1-AID Primer1	GAAGTGTATGCCATCTACGTTACTTGAACGGCGCAGCTCCTGTTATTTATGGGTTACCGATCAAGTTCGTATCACGTACGCTGCAGGTCGAC
ESP1-AID Primer2	CAAAATCGGATTTCCCATGCTTTTTCTCAATGTCTATATGAAATCTTTTCGAAACAACCAGTACATGTAACAATTAATCGATGAATTCGAGCTCG
MSH4-AID Primer1	GGAAATGAAAAAGAGCCCTTGACTTTAGGGAAATTAAAAGAAATAAACTCCGACTTCATCGAAAATTTTGAAGAACGTACGCTGCAGGTCGAC
MSH4-AID Primer2	CATTTTCTCCGGTTTTTGATAACTCTGTACAGAAATAATGGATTATAGTTTTAAGCTAAGCGGAAAAGCCAAATTAATCGATGAATTCGAGCTCG
